# Dietary epicatechin improves survival and delays skeletal muscle degeneration in aged mice

**DOI:** 10.1096/fj.201800554RR

**Published:** 2018-08-10

**Authors:** Hongwei Si, Xiaoyong Wang, Longyun Zhang, Laurence D. Parnell, Bulbul Ahmed, Tanya LeRoith, Twum-Ampofo Ansah, Lijuan Zhang, Jianwei Li, José M. Ordovás, Hongzong Si, Dongmin Liu, Chao-Qiang Lai

**Affiliations:** * Department of Human Sciences, Tennessee State University, Nashville, Tennessee, USA;; † United States Department of Agriculture (USDA) Agricultural Research Service, Jean Mayer USDA Human Nutrition Research Center on Aging at Tufts University, Boston, Massachusetts, USA;; ‡ Department of Biomedical Sciences and Pathobiology, Virginia–Maryland Regional College of Veterinary Medicine, Blacksburg, Virginia, USA;; § Department of Neuroscience and Pharmacology, Meharry Medical College, Nashville, Tennessee, USA;; ¶ Department of Agriculture and Environmental Sciences, Tennessee State University, Nashville, Tennessee, USA;; ‖ Nutrition and Genomics Laboratory, Jean Mayer USDA Human Nutrition Research Center on Aging at Tufts University, Boston, Massachusetts, USA;; # Research Institute on Food and Health Sciences, Madrid Institute of Advanced Studies (IMDEA), Campus of Universal Excellence (CEI), Autonomous University of Madrid (UAM), Madrid, Spain; ** Superior Council of Scientific Investigations (CSIC), Madrid, Spain;; †† Institute of Computational Science and Engineering, Qingdao University, Qingdao, China; and; ‡‡ Department of Human Nutrition, Foods and Exercise, Virginia Tech, Blacksburg, Virginia, USA

**Keywords:** antiaging, extracellular matrix pathway, nicotinamide pathway, PPAR pathway, lifespan

## Abstract

We recently reported that epicatechin, a bioactive compound that occurs naturally in various common foods, promoted general health and survival of obese diabetic mice. It remains to be determined whether epicatechin extends health span and delays the process of aging. In the present study, epicatechin or its analogue epigallocatechin gallate (EGCG) (0.25% w/v in drinking water) was administered to 20-mo-old male C57BL mice fed a standard chow. The goal was to determine the antiaging effect. The results showed that supplementation with epicatechin for 37 wk strikingly increased the survival rate from 39 to 69%, whereas EGCG had no significant effect. Consistently, epicatechin improved physical activity, delayed degeneration of skeletal muscle (quadriceps), and shifted the profiles of the serum metabolites and skeletal muscle general mRNA expressions in aging mice toward the profiles observed in young mice. In particular, we found that dietary epicatechin significantly reversed age-altered mRNA and protein expressions of extracellular matrix and peroxisome proliferator–activated receptor pathways in skeletal muscle, and reversed the age-induced declines of the nicotinate and nicotinamide pathway both in serum and skeletal muscle. The present study provides evidence that epicatechin supplementation can exert an antiaging effect, including an increase in survival, an attenuation of the aging-related deterioration of skeletal muscles, and a protection against the aging-related decline in nicotinate and nicotinamide metabolism.—Si, H., Wang, X., Zhang, L., Parnell, L. D., Ahmed, B., LeRoith, T., Ansah, T.-A., Zhang, L., Li, J., Ordovás, J. M., Si, H., Liu, D., Lai, C.-Q. Dietary epicatechin improves survival and delays skeletal muscle degeneration in aged mice.

Aging is a progressive, systematic dysfunction of almost all organs and tissues, as well as an escalated vulnerability to environmental challenges. The increased dysfunction and vulnerability not only result in reduced physical activity and quality of life but also compound the risks of a number of degenerative diseases such as cardiovascular disease, type 2 diabetes, cancer, Alzheimer’s disease, and sarcopenia, which account for >70% of deaths among Americans aged ≥65 yr (1). Therefore, either delaying the aging process or preventing development of these chronic diseases is an essential strategy to promote healthy aging. It is well established that calorie restriction (CR) delays age-associated organ disorders and increases life span in a wide range of species, including humans (2), suggesting that targeting nutrient-sensing and energy metabolism pathways may be an effective approach to delay the aging process and age-related diseases. However, long-term energy restriction might not be a practical strategy for the general population.

Accumulating evidence supports that food-derived small molecules (also called phytochemicals), albeit not considered essential nutrients, have the potential to extend life span in model organisms and to reduce risk of chronic diseases in humans (3, 4). Resveratrol, a well-known phytochemical, has been shown to improve health and extend life span by mimicking CR in obese mice induced by feeding of a high-fat diet (5) but not in mice fed a standard chow diet (6). These findings indicate that the resveratrol effect is mediated by favorable modulation of the increased vulnerability to the physiologic and metabolic alterations of major organs and tissues induced by feeding of a high-fat diet or obesity but not *via* directly targeting antiaging molecules within healthy aging processes. In addition, it was found that most potentially health-promoting phytochemicals such as resveratrol, green tea extract, curcumin, oxaloacetic acid, and medium-chain triglyceride oil (chain length of 6–10 carbons) all have failed to extend life span in standard diet–fed lean mice in the National Institute on Aging Interventions Testing Program (7). Therefore, identifying safe, bioactive compounds with robust antiaging effects in rodents and humans remains an elusive goal.

Cocoa intake was speculated to be the key factor that contributed to the longer and healthy life span of inhabitants of San Blas Island compared with those living on mainland Panama (8). Many studies have shown that epicatechin, a flavanol, is primarily responsible for the beneficial effects of dietary cocoa, including promoting vascular function (9) and skeletal muscle (SkM) health and combating oxidative stress (10, 11). In our screening of antidiabetic compounds, we unexpectedly found that epicatechin promoted health and survival of obese diabetic (*ob*/*ob*) mice (12). Notably, this effect of epicatechin was not the consequence of improved circulating glucose and insulin levels. We further showed that dietary provision of epicatechin extended life span in *Drosophila*. These observations suggest that epicatechin may be a novel antiaging agent. Therefore, in the present study, we investigated the effects of epicatechin supplementation on the survival rate and physical performance in aged mice fed a standard diet. In addition, transcriptome and metabolomic analyses were conducted to explore the mechanisms underlying the effects of epicatechin on survival and aging.

## MATERIALS AND METHODS

### Animals

Male C57BL/6 mice (20 or 9-mo old) were purchased from the National Institute on Aging (Bethesda, MD, USA). Mice were housed in an environmentally controlled (23 ± 2°C; 12-h light/dark cycle) animal facility and were provided free access to Teklad global rodent chow diet (Harlen, Indianapolis, IN, USA). Twenty-month-old mice were randomly divided into 3 groups (*n* = 33/group) and given either 0% [old control (OC)] or 0.25% (w/v) of epicatechin (MilliporeSigma, Burlington, MA, USA) or epigallocatechin gallate (EGCG; MilliporeSigma, Burlington, MA, USA) in drinking water for 37 wk (12 mice were euthanized to collect tissues) or 44 wk (rest of the mice were treated for 7 more wk). The 9-mo-old mice were used as young control (YC) to collect tissues only. This dosage (0.25% w/v) of epicatechin or EGCG [~150 mg/kg body weight (BW)] was calculated based on previous experiments using cocoa products in humans (100–400 mg/kg BW) and the mean concentration of flavanols in cocoa. To ensure stability of the epicatechin, stock epicatechin was stored at −80°C, and its water solution was kept from light. Fresh epicatechin solution was made and provided to the mice every other day. BW and food and water intakes were monitored weekly. Fasting (overnight) glucose was measured every 2 months, and a glucose tolerance test was conducted at month 26. The general clinical condition and mortality of the mice were monitored daily.

The criteria for euthanasia were independently assessed by a veterinarian according to the guidelines of the Association for Assessment and Accreditation of Laboratory Animal Care International. Mice with BW <30% of original BW were euthanized by inhalation of CO_2_. All natural deaths or euthanized mice were counted as death to plot the survival curves. Survival curves were plotted by using the Kaplan-Meier method and included all available animals at each time point (13). After 37 or 44 wk, the remaining mice were euthanized by inhalation of CO_2_, and blood and tissue samples including SkM (right quadriceps muscle) were immediately collected and stored at −80°C or in 10% formalin for biochemical and physiologic analysis. The animal protocol was approved by the Institutional Animal Care and Use Committee at Tennessee State University and was reported in accordance with the Animal Research: Reporting of *In Vivo* Experiments guidelines.

### Physical activity measurement and pathologic analysis

Physical activity (12 mice/group) was evaluated by performing an open field test using a computer-controlled activity monitoring system (Med Associates, St. Albans, VT, USA), as we previously reported (14). Briefly, the individual mouse was placed in the chamber for 15 min without interruption, and parameters (including distance traveled, ambulatory count, stereotypic count, time resting, vertical count, jump count, and average velocity) were recorded simultaneously. For pathologic or EGCG analysis, formalin-fixed SkM (right quadriceps muscle) samples were sectioned and stained with hematoxylin and eosin (H&E) and periodic acid–Schiff (PAS). Pathologic alterations in SkM, including extensive fiber vacuolization and degeneration, average minimum Feret diameter and central nucleation in H&E, and glycogen accumulation (the indicator of degenerative process) in PAS, were blindly evaluated as previously described (12). In brief, the pathologic alterations of quadriceps muscle were graded based on the average minimum Feret diameter (average, μm), presence of central nucleation and atrophic fibers (%) in H&E, and the accumulation of glycogen (%) in PAS. Three sections from each mouse were examined, and ten 2.37 mm^2^ fields of each section were evaluated.

### Metabolomics and metabolite analyses

Global biochemical profiles were determined by using the serum (12 mice/group) from each mouse. Metabolomic profiling analysis was performed by Metabolon (Durham, NC, USA) using ultra-high-performance liquid chromatography–tandem mass spectroscopy (UPLC-MS/MS) platforms, as previously described (15). In brief, sample (100 µl serum) preparation was conducted by using a proprietary series of organic and aqueous extractions to remove the protein fraction while allowing maximum recovery of small molecules. The resultant extract was divided into 4 fractions: one for analysis by UPLC-MS/MS (positive mode), one for UPLC-MS/MS (negative mode), one for GC-MS, and one for backup. The sample extract was dried and then reconstituted in acidic or basic liquid chromatography–compatible solvents, each of which contained ≥8 injection standards at fixed concentrations to ensure injection and chromatographic consistency. The data were extracted, with peaks and compounds identified by using the local area network backbone and database server running Oracle 10.2.0.1 Enterprise Edition (Oracle, Redwood City, CA, USA).

#### Principal component analysis

Principal components were computed by determining the coefficients of the metabolites or mRNA expression that maximize the variance of the linear combination. The total variance was defined as the sum of the variances of the predicted values of each component (the variance is the sd^2^), and for each component, the proportion of the total variance was computed.

#### Random forest

The mean decrease in accuracy was determined by randomly permuting a variable, running the observed values through the trees, and then reassessing the prediction accuracy. The result was provided as an importance rank ordering of the top 30 biochemicals.

#### Metabolic pathway analysis

All detected metabolites (*n* = 671) were organized into metabolic pathways based on the annotation database of Metabolon. Only pathways that contained ≥3 detected metabolites were included in the pathway analysis. There are a total of 58 pathways. Significantly changed metabolites between groups were selected to determine enrichment of metabolic pathways, which was determined by calculating the *z*-score test (16). Significance values were adjusted for multiple testing by using the Bonferroni test (*i.e.*, *P* ≤ 0.05/58 = 0.00086).

### mRNA sequencing and pathway analysis

Total RNA from SkM (right quadriceps) was extracted by using the RNeasy Fibrous Tissue Mini Kit (Qiagen, Valencia, CA, USA), and RNA quality was assessed by using an Agilent 2100 Bioanalyzer (Agilent Technologies, Santa Clara, CA, USA). A total of 3 μg RNA per sample was used as input material for the sequencing library preparations following the manufacturer’s protocol. The sequencing was performed by using an Illumina External HiSeq2500 High Output SE 50 (Illumina, San Diego, CA, USA). Transcriptome assembly and differential expression analyses were conducted with the Cufflinks package and default parameters (*http://cole-trapnell-lab.github.io/cufflinks/cuffdiff*). mRNA expression was expressed as fragments per kilobase of transcript per 10^6^ mapped reads (FPKM). Comparisons of gene expression between groups were performed by using the cuffdiff function within Cufflinks package. The FPKM of 8 samples of each group was used for 1- or 2-way ANOVA. Genes with an adjusted value of *P* <0.05 (*i.e.*, *q* value with false discovery rate correction for multiple testing) were assigned as significantly differentially expressed.

We selected 10,930 genes (the average FPKM value of each gene in the young control group is >1.00) in all 3 groups to conduct a principal components analysis as previously described (17). Significantly changed genes between groups were applied to the Gene Set Enrichment Analysis (*http://software.broadinstitute.org/gsea/msigdb/annotate.jsp*) using the canonical pathways to identify enriched pathways. Pathways with values of *q* < 0.5 were listed as significantly enriched pathways.

### Protein detection and measurement

To verify whether the genes with significantly differential expression result in changes in protein levels, Western blots were performed to measure relevant protein levels in SkM (right quadriceps), as we have previously described (12). Membrane fractions were probed with a primary antibody against total actin or peroxisome proliferator–activated receptor (PPAR) γ (Cell Signaling Technology, Danvers, MA, USA). Protein bands were digitally imaged for densitometric quantitation with the ImageJ software program (National Institutes of Health, Bethesda, MD, USA). The expression of the relevant protein was normalized to the glyceraldehyde-3-phosphate dehydrogenase level from the same sample.

### Statistical analyses

Survival curves were plotted by using the Kaplan-Meier method, including all available mice at each time point, and the log-rank test was applied to compare the survival distributions of the control and epicatechin-treated (EC) groups. The results from pathologic analysis of the SkM were analyzed by using a Kruskal-Wallis test. All other data were analyzed with 1-way ANOVA, and significant differences between treatment groups were further analyzed by using the Tukey test. Values of *P* < 0.05 were considered statistically significant.

## RESULTS

### Epicatechin supplementation increases survival of aged mice

**Figure 1:**
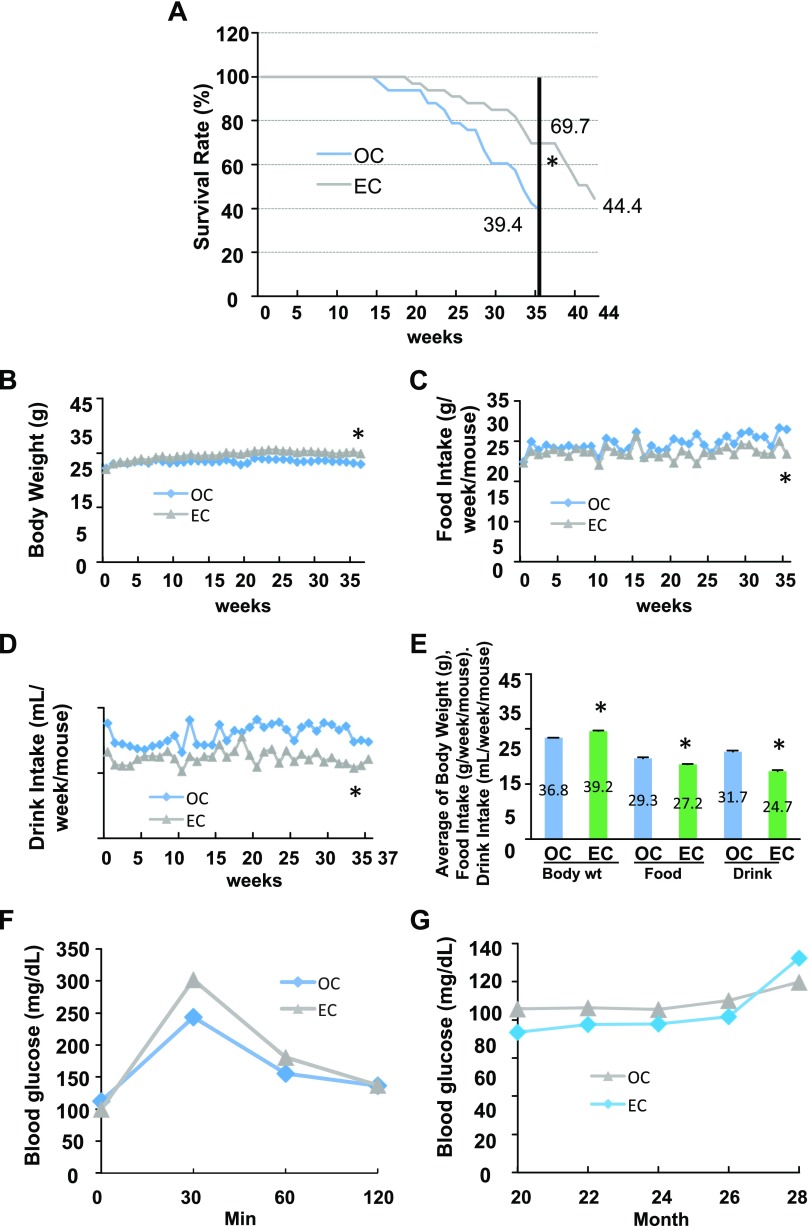
Effects of dietary supplementation of epicatechin on survival rate in aged mice fed a standard diet. Twenty-month-old male C57BL/6 mice were treated with EC (0.25% w/v in drinking water) or control (OC) for 37 or 44 wk (*n* = 33/group). *A*) Epicatechin intake significantly increased survival rate of mice. *B–D*) Epicatechin intake enhanced BW (*B*) while decreasing food and drink intake (*C*, *D*) during the experimental period. *E*) The average BW, food intake, and drink intake of the entire experimental period. *F, G*) Epicatechin intake did not significantly affect the results of the glucose tolerance test (*F*) or fasting plasma glucose levels (*G*). *P < 0.05 EC *vs.* OC (*n* = 12–33).

To determine the effects of epicatechin on life span, 20 mo old C57BL/6 male mice fed the standard diet were provided with epicatechin or EGCG dissolved in drinking water (0.25% w/v) for 37–44 wk. We observed that old mice given epicatechin intake had a strikingly higher survival rate (69%) compared with the OC mice (39%) after 37 wk of treatment (**Fig. 1*A***). In total, 44% of mice were still alive in the EC group after 7 additional wk of epicatechin treatment. However, EGCG supplementation had no significant antiaging effect (*P* = 0.56) as the survival curves of EGCG and OC were almost overlapped before wk 29 after removing the first 5 dead mice because of EGCG supplementation–induced dehydration (Supplemental Fig. 1). Therefore, this dehydration may mask, enhance, or otherwise alter the EGCG antiaging effects. Epicatechin-supplemented mice slightly gained BW (Fig. 1*B*) and had reduced food and water intakes compared with the OC group (Fig. 1*C, D*). These patterns of the average BW and food and drink intake are in line with the data of these measurements over the entire experimental period (Fig. 1*E*). Interestingly, the amount of visceral fat was significantly higher in EC mice compared with the OC group (Supplemental Table 1). However, epicatechin did not significantly alter glucose tolerance (Fig. 1*F*) and fasting blood glucose (Fig. 1*G*) in aged mice, in agreement with our previous report (12); these findings suggest that the epicaechin life span–extending effect in aged mice is not *via* regulation of glucose metabolism.

### Epicatechin increases physical activity levels and delays SkM degeneration

**Figure 2:**
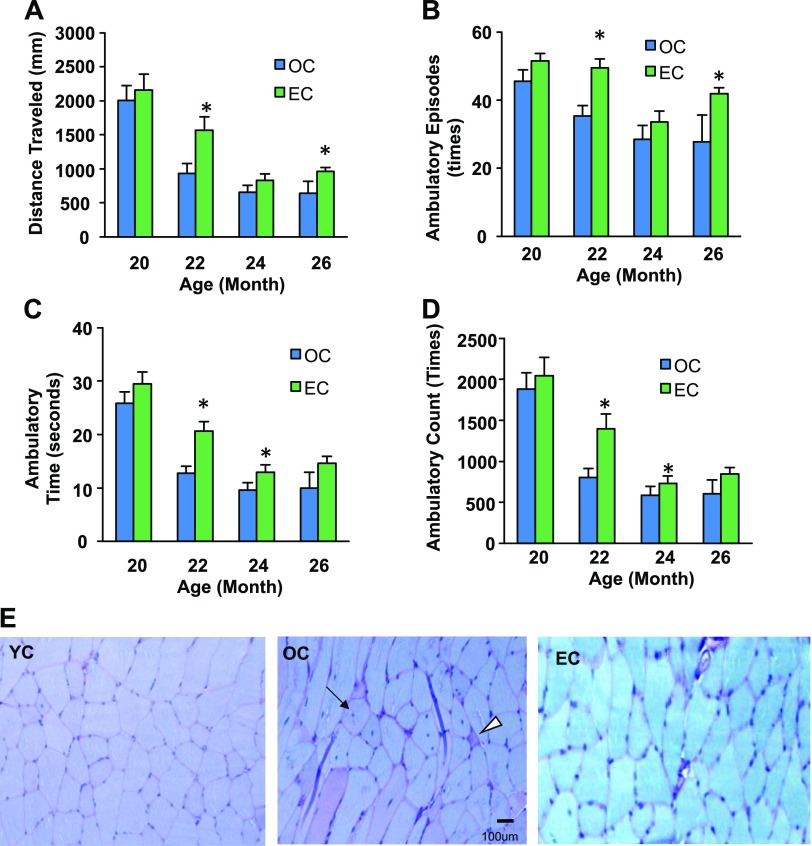
Effects of dietary epicatechin on physical activity and SkM pathology. Twenty-month-old male C57BL/6 mice were treated with EC (0.25% in drinking water) or control (OC) for 9 mo (37 wk). *A–D*) Physical activity metrics [distance traveled (*A*), ambulatory episodes (*B*), ambulatory time (*C*), ambulatory count (*D*)] were tested by using a computer-controlled activity monitoring system at 20, 22, 24, and 28 mo of age (*n* = 12/group). *E*) SkM tissue (quadriceps) specimens were pathologically analyzed after H&E and PAS staining. Original magnification, ×400: Arrow indicates central nucleation; arrowhead indicates degenerating fiber. ^#^*P* < 0.05 OC *vs.* YC; *P < 0.05 EC *vs.* OC. *n* = 12.

Because a decline in physical activity due to dysfunction of SkM is associated with aging, we measured physical activity of the mice throughout the experiment by using a computer-controlled monitoring system (14). Epicatechin intake improved physical activity metrics, including traveling distance (a measurement of gross locomotor activity) (**Fig. 2*A***) and the time, counts, and episodes of ambulatory (exploratory behavior) in aged C57BL/6 mice (Fig. 2*B–D*).

Age-related dysfunction of SkM is associated with a reduction of protein synthesis and an increase in protein degradation (18). To determine whether epicatechin delays SkM degradation, a pathologic analysis of SkM was conducted. As shown in Fig. 2*E*, OC mice displayed a dramatic increase in SkM degeneration characterized by extensive fiber vacuolization and degeneration (central nucleation and degenerated fibers) and accumulation of glycogen in some atrophic fibers compared with the YC mice. However, epicatechin intake significantly ameliorated these age-associated degenerative changes of SkM in aged mice, indicating that dietary epicatechin inhibits the age-associated dysfunction of SkM.

### Epicatechin reverses age-induced metabolomic changes associated with the NAD metabolic pathway

**Figure 3:**
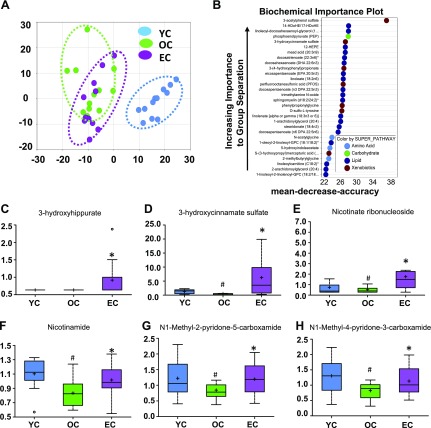
Dietary epicatechin reversed age-related metabolomic changes associated with NAD pathway in aged mice. Principal component analysis revealed perfect separation between YC and OC, and EC intake significantly shifted back to YC (*A*). Random forest analysis resulted in a predicted accuracy of 78% between groups (*B*), and levels of 6 metabolites were significantly changed by EC compared with the OC group (*C–H*). ^#^*P* < 0.05 EC *vs.* YC; *P < 0.05 EC *vs.* OC (*n* = 12/group).

A metabolomic analysis of serum was conducted to determine the difference in metabolic profiles of YC, OC, and EC mice. A total of 671 metabolites were detected in the serum, with levels of 390 being significantly different (139 reduced, 251 increased) between young and aged mice. Among these, 47 metabolite levels were altered by EC treatment in aged mice. Principal component analysis based on all 671 metabolites revealed perfect separation between YC and OC mice, and epicatechin supplementation significantly shifted the metabolic profiles of old mice toward those in young mice (**Fig. 3*A***). Using the detected 671 metabolites, a further random forest analysis was performed to identify 36 metabolites that distinguish the 3 groups (YC, OC, and EC). This method resulted in a predicted accuracy of 78% between groups using these 36 metabolites (Fig. 3*B*). The top 2 categories of metabolites identified according to the random forest analysis were lipids and xenobiotics, indicating that these metabolites could serve as biomarkers of the epicatechin antiaging effects. The 6 metabolites that were most significantly different between the OC and EC groups are 3-hydroxyhippurate, 3-hydroxycinnamate sulfate, nicotinate ribonucleoside, nicotinamide, N1-methyl-2-pyridone-5-carboxamide, and N1-methyl-4-pyridone-3-carboxamide (Fig. 3*C–H*).

NAD^+^ is an essential electron transporter in mitochondrial respiration and oxidative phosphorylation (19), and NAD metabolism is important in aging processes. Interestingly, aging reduced serum levels of 2 major NAD metabolism-related molecules—nicotinate ribonucleoside (Fig. 3*E*) and nicotinamide (Fig. 3*F*)—but these reductions were reversed by epicatechin. Furthermore, our metabolic pathway analysis identified that the NAD metabolic pathway (*P* = 0.00081) is one of the top 5 pathways whose metabolite components are significantly different between OC and EC mice after correction of multiple testing (**Table 1**). These findings support an important role for NAD in the antiaging effect of epicatechin, given that NAD^+^ levels decline with age (20).

**TABLE 1:** Enriched metabolic pathways that are associated with epicatechin supplementation

Pathway	Metabolite (*n*)^*a*^	EC *vs.* OC (*P* ≤ 0.05)^*b*^	*Z* score	*P*^*c*^
Benzoate metabolism	16	5	4.145	3.40E−05
Glycine, serine, and threonine metabolism	9	3	3.348	8.14E−04
Pyrimidine metabolism, uracil containing	9	3	3.348	8.14E−04
NAD metabolism	9	3	3.348	8.14E−04
Glycolysis, gluconeogenesis, and pyruvate metabolism	8	2	2.18	2.93E−02
Pyrimidine metabolism, cytidine containing	9	2	1.97	4.88E−02

a Detected metabolites assigned to the respective pathway. *^b^*Metabolites that are significantly different between EC and OC mice. *^c^*Underlined values are significant after correction for multiple testing by using the Bonferroni test (*i.e.*, *P* ≤ 0.00086).

### Epicatechin reverses age-related changes in genes associated with the extracellular matrix and PPAR pathways

**Figure 4:**
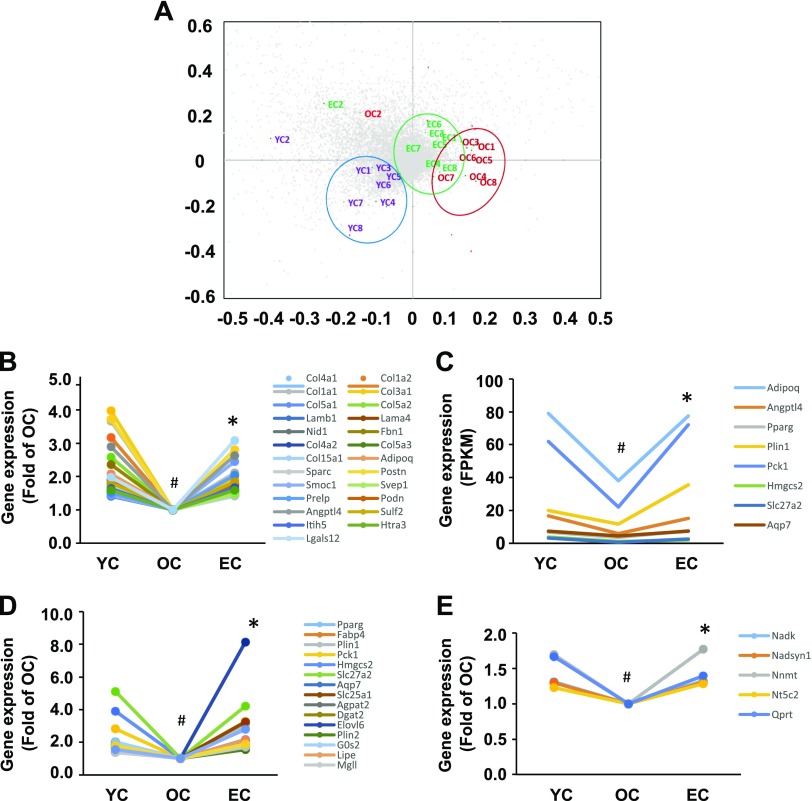
Dietary epicatechin reversed age-related changes in gene expressions and pathways of quadriceps muscles in aged mice. The mRNA sequencing results show that a clear separation between YC and OC, and epicatechin intake significantly shifted back to YC (*A*) according to the principal component analysis. Nearly all genes of 4 pathways—matrisome [25 genes (*B*)], PPAR [8 genes (*C*)], lipids and lipoprotein [17 genes (*D*)], and NAD [5 genes (*E*)]—showed decreased expression during aging, but expression of all genes were significantly reversed by epicatechin intake. ^#^*P* < 0.05 EC *vs.* YC; *P < 0.05 EC *vs.* OC (*n* = 8/group).

Because epicatechin intake improved SkM function and curtailed SkM pathologic degeneration, we then performed transcriptome analysis by conducting whole genome RNA sequencing of the SkM tissue. We selected 10,930 genes (whereby the average FPKM value of each gene in the YC group was >1.00) to conduct a principal components analysis to characterize the differences between the 3 groups. Dietary supplementation of epicatechin significantly shifted the transcriptome profiles of old mice toward those in young mice (**Fig. 4*A***), indicating that epicatechin intake reversed age-associated changes in SkM gene expression. We found that 791 genes in OC mice displayed significant expression changes (599 decreased, 192 increased) compared with YC mice, and 222 genes in EC mice were significantly different compared with OC mice. Further analysis revealed that mRNA levels of 162 genes were reduced in OC mice compared with YC mice but were reversed by epicatechin intake. Among these 2 sets of differential expression genes (791 genes of YC *vs.* OC, and 222 genes of EC *vs.* OC), we observed that 92 transcripts were common. Of these 92 genes, expression levels of 87 were significantly reduced in OC mice compared with YC mice; they were significantly reversed in the EC group, however, underscoring the antiaging effect of epicatechin in SkM.

The differentially expressed genes were then subjected to Gene Set Enrichment Analysis with canonical pathways to identify the pathways associated with epicatechin antiaging effects. By applying those genes with significant changes in their expression levels between YC and OC, OC and EC, or shared genes between 2 sets (YC *vs.* OC and OC *vs.* EC), and correcting for multiple testing, a set of pathways were identified that were modulated by epicatechin treatment (**Table 2** and Supplemental Table S2) and by aging (Table 2 and Supplemental Table S3). In particular, we found that extracellular matrix (ECM), PPAR-signaling pathway, and metabolism of lipid and lipoprotein are the top 4 pathways in all 3 comparisons (Table 2 and Supplemental Tables S2–4), and these pathways play critical roles in SkM function (21, 22). Our subsequent analysis indicated that mRNA expression of most genes in the ECM (24 genes) (Fig. 4*B*), *PPAR* (8 genes) (Fig. 4*C*), and lipid and lipoprotein (17 genes) (Fig. 4*D*) pathways were reduced in old mice compared with those in young mice but were significantly reversed by epicatechin intake. Particularly, 5 gene transcripts in the NAD pathway were significantly reduced in old mice relative to the young mice but were up-regulated by epicatechin to the levels comparable to those of the young mice (Fig. 4*E*), which matches the effect of epicatechin on serum NAD-related molecule levels (Fig. 3). These results indicate that the antiaging effect of epicatechin is likely mediated *via* these 4 pathways.

**TABLE 2:** Top 10 enriched gene sets (pathways) that are associated with aging and epicatechin supplementation in SkM

Gene	Gene set name (pathway)*^a^*	*K*	Gene description	*k*	*k*/*K^b^*	*P*	FDR *q*
YC *vs.* OC (791 genes applied)*^c^*	NABA_MATRISOME	1028	Ensemble of genes encoding ECM and ECM-associated proteins	137	0.1333	9.44E−85	1.25E−81
	NABA_CORE_MATRISOME	275	Ensemble of genes encoding core ECM, including ECM glycoproteins, collagens, and proteoglycans	68	0.2473	2.00E−60	1.33E−57
	NABA_ECM_GLYCOPROTEINS	196	Genes encoding structural ECM glycoproteins	42	0.2143	7.28E−35	3.22E−32
	NABA_MATRISOME_ASSOCIATED	753	Ensemble of genes encoding ECM-associated proteins, including ECM-affiliated proteins, ECM regulators, and secreted factors	69	0.0916	6.64E−32	2.21E−29
	NABA_ECM_REGULATORS	238	Genes encoding enzymes and their regulators involved in the remodeling of the ECM	39	0.1639	8.52E−28	2.26E−25
	KEGG_COMPLEMENT_AND_COAGULATION_CASCADES	69	Complement and coagulation cascades	25	0.3623	1.24E−27	2.35E−25
	KEGG_PPAR_SIGNALING_PATHWAY	69	PPAR-signaling pathway	25	0.3623	1.24E−27	2.35E−25
	REACTOME_METABOLISM_OF_LIPIDS_AND_LIPOPROTEINS	478	Genes involved in metabolism of lipids and lipoproteins	51	0.1067	7.24E−27	1.20E−24
	KEGG_FOCAL_ADHESION	201	Focal adhesion	35	0.1741	5.28E−26	7.80E−24
	PID_INTEGRIN1_PATHWAY	66	Beta_1_-integrin cell surface interactions	21	0.3182	4.81E−22	6.39E−20
OC *vs.* EC (222 genes applied)*^d^*	NABA_CORE_MATRISOME	275	Ensemble of genes encoding core ECM, including ECM glycoproteins, collagens, and proteoglycans	20	0.0727	1.58E−18	2.10E−15
	NABA_MATRISOME	1028	Ensemble of genes encoding ECM and ECM-associated proteins	29	0.0282	3.03E−15	2.01E−12
	REACTOME_DEVELOPMENTAL_BIOLOGY	396	Genes involved in developmental biology	19	0.048	2.38E−14	1.05E−11
	REACTOME_METABOLISM_OF_LIPIDS_AND_LIPOPROTEINS	478	Genes involved in metabolism of lipids and lipoproteins	20	0.0418	6.23E−14	2.07E−11
	NABA_COLLAGENS	44	Genes encoding collagen proteins	9	0.2045	3.47E−13	8.96E−11
	PID_INTEGRIN1_PATHWAY	66	Beta_1_-integrin cell surface interactions	10	0.1515	4.04E−13	8.96E−11
	KEGG_PPAR_SIGNALING_PATHWAY	69	PPAR-signaling pathway	10	0.1449	6.44E−13	1.22E−10
	REACTOME_NEURONAL_SYSTEM	279	Genes involved in neuronal system	15	0.0538	2.75E−12	4.44E−10
	REACTOME_TRANSMISSION_ACROSS_CHEMICAL_SYNAPSES	186	Genes involved in transmission across chemical synapses	13	0.0699	3.01E−12	4.44E−10
	KEGG_ECM_RECEPTOR_INTERACTION	84	ECM–receptor interaction	10	0.119	4.94E−12	5.97E−10
Both YC *vs.* OC and OC *vs.* EC (92 genes applied)*^e^*	NABA_CORE_MATRISOME	275	Ensemble of genes encoding core ECM, including ECM glycoproteins, collagens, and proteoglycans	18	0.0655	1.14E−22	1.52E−19
	NABA_MATRISOME	1028	Ensemble of genes encoding ECM and ECM-associated proteins	23	0.0224	2.51E−18	1.67E−15
	PID_INTEGRIN1_PATHWAY	66	Beta_1_-integrin cell surface interactions	10	0.1515	8.50E−17	3.76E−14
	KEGG_ECM_RECEPTOR_INTERACTION	84	ECM–receptor interaction	10	0.119	1.08E−15	3.59E−13
	REACTOME_DEVELOPMENTAL_BIOLOGY	396	Genes involved in developmental biology	15	0.0379	1.76E−15	4.68E−13
	NABA_COLLAGENS	44	Genes encoding collagen proteins	8	0.1818	2.40E−14	5.31E−12
	KEGG_FOCAL_ADHESION	201	Focal adhesion	11	0.0547	2.31E−13	4.22E−11
	REACTOME_COLLAGEN_FORMATION	58	Genes involved in collagen formation	8	0.1379	2.54E−13	4.22E−11
	KEGG_PPAR_SIGNALING_PATHWAY	69	PPAR-signaling pathway	8	0.1159	1.09E−12	1.56E−10
	REACTOME_NCAM1_INTERACTIONS	39	Genes involved in NCAM1 interactions	7	0.1795	1.17E−12	1.56E−10

FDR, false discovery rate; *K*, genes in gene set (*n*); *k*, genes shared (*n*); NCAM, neural cell adhesion molecule. *^a^*Associated pathways with differential gene expressions were identified by using Gene Set Enrichment Analysis (*http://software.broadinstitute.org/gsea/msigdb*). The list of top 100 pathways is provided in Supplemental Tables 2–4. *^b^k*/*K* = proportion of identified differential expression genes for a given gene set (pathway). *^c^*Top 10 enriched pathways that were associated with aging in SkM. *^d^*Top 10 enriched pathways that were associated with epicatechin supplementation in SkM of OC mice. *^e^*Top 10 enriched pathways that were associated with aging and epicatechin supplementation in SkM.

Epicatechin also reversed the age-related changes in expression of select genes involved in fatty acid metabolism (Table 2). The expression of *Hacd2*, or *Ptplb*, which encodes the major 3-hydroxyacyl-CoA dehydratase in SkM (23), was >1-fold lower in OC mice than in YC mice but was normalized in EC mice (2.2, 0.98, and 2.4 FPKM), respectively. Similarly, *Fabp4*, which is involved in cellular fatty acid uptake, transport, and metabolism, exhibited FPKM values of 732, 386, and 672 FPKM in YC, OC, and EC; *Acaca*, encoding the enzyme responsible for the rate-limiting step of fatty acid synthesis, had FPKM values of 7.3, 6.1, and 14.5 in YC, OC, and EC mice. Lastly, dietary epicatechin treatment more than compensated for the age-dependent declines in the expression of *Slc1a3*, encoding a glutamate transporter, and *Slc25a1*, encoding a mitochondrial citrate transporter, with expression values of 4.0, 2.4, and 5.5 FPKM, and 5.4, 3.8, and 12.4 FPKM in YC, OC, and EC mice.

Myokines are important agents that regulate metabolic responses and act as autocrine, endocrine, and/or paracrine factors in SkM (24). Our analysis of gene expression in SkM from old mice on the control diet compared with EC old mice indicated 4 myokine-encoding genes with statistically significant altered expression: *Adipoq*, *Angptl4*, *S100a8*, and *Sparc*. *Adipoq*, *Angptl4*, and *S100a8* increased significantly in SkM from mice on EC supplementation 2.04-, 2.63-, and 9.39-fold, respectively (*q* = 0.0044 for all). Levels of *Sparc* mRNA were noted to increase 1.42-fold (*q* = 0.014) on the supplemented diet.

### Epicatechin reverses age-associated changes of protein expression in SkM

**Figure 5:**
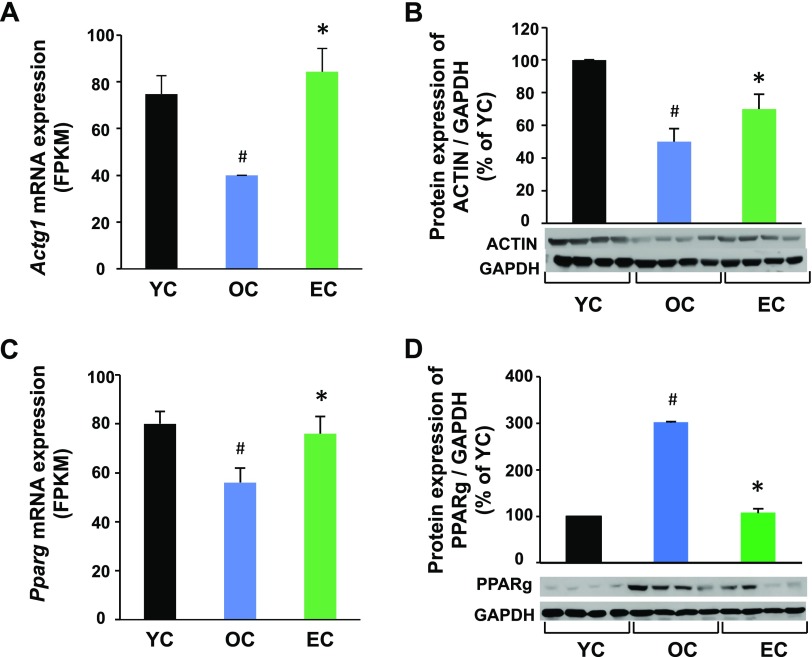
Dietary epicatechin reversed age-associated protein expressions in SkM of aged mice. Skeletal muscle samples (quadriceps) were collected at 37 wk in the EC and OC groups or at 12 mo of age in the YC group (*n* = 12/group). Total actin (all 6 isoforms) (*B*) or PPARγ (*D*) protein expression was measured by Western blot and normalized to glyceraldehyde-3-phosphate dehydrogenase (GAPDH) content. The mRNA expression of actin (*A*) or PPARγ (*C*) was obtained from the RNA sequencing as described in Fig. 4. ^#^*P* < 0.05 EC *vs.* YC, *P < 0.05 EC *vs.* (*n* = 8/group).

To determine whether the altered mRNA levels correlated with their corresponding protein expression, we measured several proteins whose transcripts were significantly changed by aging and were reversed by epicatechin in SkM. Both actin mRNA and protein expression were reduced in OC mice compared with YC mice, but epicatechin intake significantly reversed both actin mRNA and protein expression (**Fig. 5*A, B***). However, PPARγ mRNA expression was opposite to its protein expression in aged mice. As shown in Fig. 5*C*, PPARγ mRNA expression was significantly reduced in OC mice compared with YC mice, but PPARγ protein expression was increased by 3-fold in the OC group compared with the YC group (Fig. 5*D*), showing that protein expression does not always correspond perfectly with its mRNA expression. Interestingly, epicatechin intake reversed the age-altered PPARγ mRNA and protein expression in OC mice to levels similar to those in the YC group, indicating that the response is maintained by epicatechin intake both in mRNA and protein levels.

## DISCUSSION

There has been considerable interest in identifying natural compounds that have the capacity to promote health and extend life span. Polyphenolic compounds, including resveratrol (25), green tea extract and its major bioactive component EGCG (26), curcumin (27), and quercetin (28), have been shown to extend life span in various model organisms such as yeast, roundworms, fruit flies, and obese mice. The National Institute on Aging Interventions Testing Program, however, reported that the natural compounds that presumably have potential to exert life-extension effects, including resveratrol, green tea extract, curcumin, oxaloacetic acid, and medium-chain triglyceride oil, all failed to extend life span in standard diet–fed lean mice (7). This outcome suggests that those previously identified antiaging compounds exert an antiaging effect only under certain pathologic conditions such as obesity and metabolic syndrome induced by long-term feeding of a high-fat diet. In the present study, we present 4 lines of evidence (survival rate, gene/and protein expression and pathology in SkM, and serum metabolites) showing that epicatechin supplementation exerts antiaging effects in aged lean mice fed a standard diet. Hence, the present study is important in antiaging research of natural compounds because epicatechin exerted its antiaging effects in normal aging mice without manipulation of their diet. In line with improved health and prolonged life span, epicatechin intake improved aging-induced SkM degeneration and reversed aging-altered SkM mRNA and protein expression of several key components of the ECM and PPAR pathways, as well as the serum metabolomic profiles and the NAD pathway in old mice. Moreover, EGCG, the major bioactive compound of green tea, did not significantly increase the survival rate in aging mice in this study (data not shown), indicating epicatechin is a special phytochemical eliciting antiaging effects. Together, these findings suggest that epicatechin may be a novel food-derived antiaging agent, acting in normal aging subjects at least partially by directly exerting protective effects on SkM.

Progressive loss of SkM mass and muscle strength, a condition known as sarcopenia, is one of the most notable and debilitating alterations associated with the aging process. Sarcopenia is noted in a wide range of organisms, from nematodes, flies, rodents, and nonhuman primates to humans (29). This loss of muscle mass and strength results in the reduced capacity for endurance and increased muscle wasting and fatigability, and therefore reduced capacity for physical activity (30). In the present study, we found that the effects of epicatechin are accompanied by an improvement in age-related decline of physical activity and a reversal of age-induced SkM pathologic changes. These data are supported by observations that epicatechin reversed aging-associated changes in transcriptome profiles and protein expression of key elements of the ECM and PPAR pathways in SkM (Fig. 4). These results suggest that dietary epicatechin may delay aging *via* maintaining organ physiology and thereby normal function. Interestingly, exercise (31), CR (32), or combined exercise and CR (33) can attenuate age-induced SkM dysfunction, particularly through the PPAR-mediated suppression of inflammation. Therefore, epicatechin intake could mimic the antiaging and antisarcopenia effects of exercise and CR.

Myokines were examined for epicatechin-induced changes in expression as an additional level of evidence that the observed effects of diet supplementation act on SkM in a manner that permits a broad range of effects with implications on muscle function. Four myokines showed increased expression with epicatechin supplementation. Adiponectin, encoded by *Adipoq*, has central metabolic and insulin-sensitizing effects on SkM (34). Similarly, *Adipoq* expression was increased in SkM of mice after 10 wk of endurance constant-moderate intensity exercise with concomitant increase of expression of *Ppargc1a* and *Ucp2* (35). Several roles have been ascribed to Angptl4, including triglyceride trafficking into peripheral tissues for use as fuel (36), notably in active SkM (24), and a protective role against muscle atrophy (37). Expression of myokine *S100a8* increased in muscle after exercise (38) and in fasting state human blood was correlated with lactate levels at rest (39). Human *SPARC* was up-regulated in SkM after exercise training regardless of age or type of exercise, with an assigned role in angiogenesis (40). For these 4 myokines, the epicatechin-stimulated increases in expression mirror expression values observed in YC samples.

Data from a serum metabolomic analysis showed that dietary supplementation of epicatechin significantly shifted the metabolic profiles of old mice toward those in young mice (Fig. 3*A*). Notably, epicatechin restored the down-regulated NAD metabolic pathway, which could primarily mediate the epicatechin antiaging effect (Table 1). NAD^+^ is an essential electron transporter in mitochondrial respiration and oxidative phosphorylation. It was found that NAD^+^ levels decline with age due to increased NAD^+^ catabolism in rodent and human tissues (19), and maintaining NAD^+^ levels could reverse age-related multiple disorders in old mice (20). We observed that circulating levels of 2 NAD metabolites (nicotinate ribonucleoside and nicotinamide) were significantly reduced in old mice but were restored by epicatechin (Fig. 3*E, F*). Consistently, 5 NAD-related gene transcripts (*Nadk*, *Nadsyn1*, *Nnmt*, *Nt5c2*, and *Qprt*) declined during aging in SkM but were reversed by treatment with epicatechin (Fig. 4*E*). These results corroborate a study that dietary cocoa polyphenol augmented intracellular NAD^+^ pools in diabetic rats (41). Collectively, these facts suggest that the epicatechin antiaging effect may be at least partially mediated by NAD metabolism.

The SkM ECM, a collection of extracellular molecules secreted by cells that accounts for ∼10% of muscle weight, is vital in tissue development, structural support, and force transmission. The major molecules of ECM in SkM are: *1*) type I, III, and VI collagens and fibronectin located in the outer reticular lamina; and *2*) collagen type IV and laminin located in the inner basal lamina (42). Structural and biologic changes, including increased collagen levels and decreased collagen turnover with accumulation of enzymatically mediated collagen cross-links, contribute to the deterioration in muscle mechanical properties with aging (43). It has been reported that ECM mediates the SkM aging process in seals (44), mice (21), and rats (45) by disrupting muscle stem cell function (46). In the present study, ECM-related gene sets dominate the top aging-sensitive gene sets (4 of 10 top gene sets) comparing young mice, old mice, and old mice treated with epicatechin. Most importantly, 24 genes (including type I, III, IV and VI collagens, laminin, and fibrillin) of the Matrisome gene set (ensemble of 1028 genes encoding ECM and ECM-associated proteins) were reduced in aged mice but were significantly reversed by epicatechin intake, confirming that epicatechin has the ability to reverse an important aspect of SkM aging. Thus, the epicatechin antiaging effects at least can be mediated by altered ECM in the aging SkM.

We found that PPAR, the nuclear hormone receptor superfamily of transcription factors, is the second top molecule contributing to the epicatechin-induced changes of SkM gene expression. The transcription of 9 genes was significantly reduced in old mice compared with young mice, but EC intake significantly reversed the expression of all 9 genes (Fig. 3*A*), indicating that this signaling pathway, particularly PPARγ, may mediate the protective effect of EC in SkM. In fact, PPARγ is expressed in a range of tissues, including adipocytes, SkM cells, osteoclasts, and others, which itself suggests that dietary EC also affects other tissues. PPARγ plays a vital role in a broad spectrum of biologic processes (47), and it is an important anti-inflammatory factor (48). It has been shown that inflammation is a major cause of sarcopenia (22). Aging is associated with increased low-grade, chronic inflammation accompanied by reduced PPARγ activity and expression (32), whereas exercise (31), CR (32), and combined exercise and CR (33) can reverse the alterations of PPARγ and inflammation, thereby attenuating age-induced SkM dysfunction. Our results indicate that EC intake might mimic the effects of exercise and CR on the anti-inflammation and PPARγ by reversing age-altered PPARγ protein and gene expressions in SkM (Fig. 5*C, D*). Moreover, we found that the mRNA levels of *Nfkb2*, encoding NFκB, was significantly increased in SkM of old mice compared with that in young mice. However, EC almost completely reversed the abnormally increased expression of *Nfkb2* in SkM of old mice. Because the anti-inflammatory effect of PPARγ is mediated *via* inhibiting NF-κB transcriptional activity in SkM (49), the effect of EC on *Nfkb2* might be secondary, and thereby EC up-regulated PPARγ. Nevertheless, the antiaging effects of EC may be partially due to its anti-inflammatory action mediated by PPARγ.

In support of the epicatechin-directed gene expression changes in components of the PPARγ-signaling pathway and its role in fatty acid metabolism, we observed 3 genes involved in this metabolic process (*Acaca*, *Fabp4*, and *Hacd2*) whose expression in SkM of old mice was significantly changed compared with that in the SkM of YC mice. These expression changes also completely reversed the observed age-related declines in mRNA counts. Because increased accumulation of lipid content in aging SkM could cause progressive sarcopenia (30), the mRNA profile of these genes as induced by dietary epicatechin is noteworthy. In addition, epicatechin-reversed expression of the high-affinity glutamate transporter *Slc1a3*, or *EAAT1*, was shown to promote resistance to hypoxic injury in astrocyte-neuron cocultures (50); this finding may be relevant to cellular processes of the neuromuscular junction (51) and hence overall SkM function.

There are several limitations of the present study. First, the mice were euthanized when the control group had 12 mice left; a complete Kaplan-Meier survival curve is needed for this antiaging study. Second, only a single dose of pure epicatechin (0.25% w/v) was used; this dosage is very high and very difficult to achieve by dietary intake of foods containing epicatechin. Thus, a dose-dependent study is needed to assess whether a lower dosage has similar effects. Third, it is unclear from this study whether EC has a similar life span– extending effect in female mice, as sex could play an important role in aging dynamics (52). Naturally, this epicatechin antiaging effect also may result from the impact on other organs such as the brain, liver, heart, and spleen.

In summary, to the best of our knowledge, we have shown for the first time that the dietary intake of epicatechin significantly increased the survival rate of aged normal mice fed a standard diet. This antiaging effect of epicatechin is associated with improvements in physical activity and changes in serum metabolite and SkM transcription profiles toward those of YC mice. Particularly, epicatechin intake abrogated the age-induced down-regulation of the NAD pathway both in serum and SkM as well as in ECM and PPAR pathways in SkM. Given that epicatechin exerts these effects in aging mice without modulation of their diet, it could be a potential food-derived small molecule capable of prolonging a healthy life span.

## Supplementary Material

This article includes supplemental data. Please visit *http://www.fasebj.org* to obtain this information.

## Abbreviations

BW: body weight
CR: calorie restriction
EC: epicatechin-treated
ECM: extracellular matrix
EGCG: epigallocatechin gallate
FPKM: fragments per kilobase of transcript per 10^6^ mapped reads
H&E: hematoxylin and eosin
PPAR: peroxisome proliferator–activated receptor
NAD: nicotinate and nicotinamide
OC: old control
PAS: periodic acid–Schiff
SkM: skeletal muscle
UPLC-MS/MS: ultra-high-performance liquid chromatography–tandem mass spectroscopy
USDA: U.S. Department of Agriculture
YC: young control
