# Supplementary material for: Dietary epicatechin improves survival and delays skeletal muscle degeneration in aged mice
Source: FASEB J. 2018 Aug 10;33(1):965–77. doi: 10.1096/fj.201800554RR (PMC6355074; doi:10.1096/fj.201800554RR)
Supplement: Supplementary file 1 [file fj.201800554RR.sd1.pptx]

## Slide 1
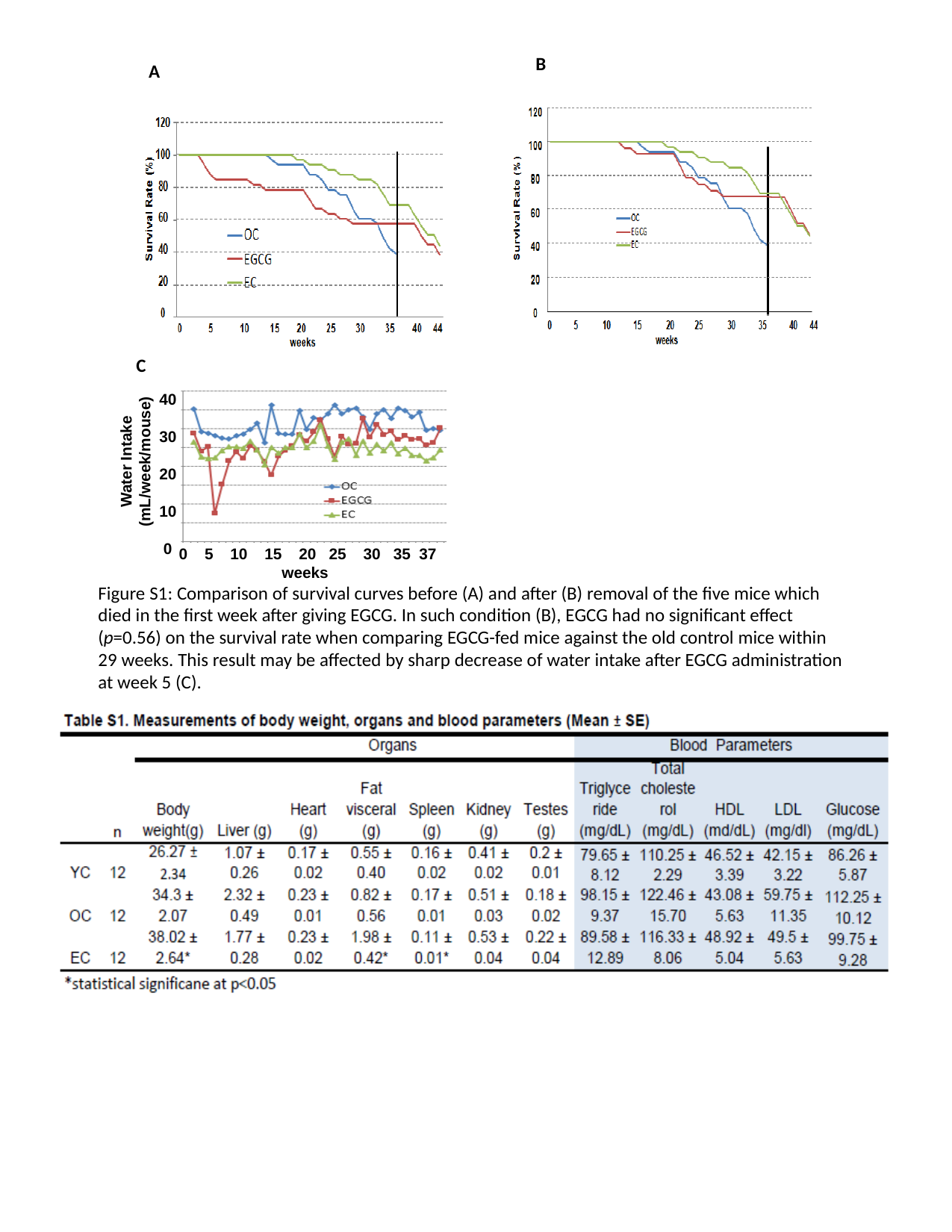

B
A
C
40
30
20
10
0
Water Intake (mL/week/mouse)
0 5 10 15 20 25 30 35 37
 weeks
Figure S1: Comparison of survival curves before (A) and after (B) removal of the five mice which died in the first week after giving EGCG. In such condition (B), EGCG had no significant effect (p=0.56) on the survival rate when comparing EGCG-fed mice against the old control mice within 29 weeks. This result may be affected by sharp decrease of water intake after EGCG administration at week 5 (C).
