# Supplementary material for: Dietary epicatechin improves survival and delays skeletal muscle degeneration in aged mice
Source: FASEB J. 2018 Aug 10;33(1):965–77. doi: 10.1096/fj.201800554RR (PMC6355074; doi:10.1096/fj.201800554RR)
Supplement: Supplementary file 2 [file fj.201800554RR.st2.pdf]

**Supplemental table 2: Top 100 pathways that were associated with aging based on 791 genes with significantly differential expression between YC and OC (i.e., YC vs. OC).**

| Gene Set (Pathway) Name | # Genes in Gene Set (K) | Description | # Genes in Overlap (k) | k/K | p-value | FDR q-value |
|---|---|---|---|---|---|---|
| NABA_MATRISOME | 1028 | Ensemble of genes encoding extracellular matrix and extracellular matrix-associated proteins | 137 | 0.1333 | 9.44E-85 | 1.25E-81 |
| NABA_CORE_MATRISOME | 275 | Ensemble of genes encoding core extracellular matrix including ECM glycoproteins, collagens and proteoglycans | 68 | 0.2473 | 2.00E-60 | 1.33E-57 |
| NABA_ECM_GLYCOPROTEINS | 196 | Genes encoding structural ECM glycoproteins | 42 | 0.2143 | 7.28E-35 | 3.22E-32 |
| NABA_MATRISOME_ASSOCIATED | 753 | Ensemble of genes encoding ECM-associated proteins including ECM-affilaited proteins, ECM regulators and secreted factors | 69 | 0.0916 | 6.64E-32 | 2.21E-29 |
| NABA_ECM_REGULATORS | 238 | Genes encoding enzymes and their regulators involved in the remodeling of the extracellular matrix | 39 | 0.1639 | 8.52E-28 | 2.26E-25 |
| KEGG_COMPLEMENT_AND_COAGULATION_CASCADES | 69 | Complement and coagulation cascades | 25 | 0.3623 | 1.24E-27 | 2.35E-25 |
| KEGG_PPAR_SIGNALING_PATHWAY | 69 | PPAR signaling pathway | 25 | 0.3623 | 1.24E-27 | 2.35E-25 |
| REACTOME_METABOLISM_OF_LIPIDS_AND_LIPOPROTEINS | 478 | Genes involved in Metabolism of lipids and lipoproteins | 51 | 0.1067 | 7.24E-27 | 1.20E-24 |
| KEGG_FOCAL_ADHESION | 201 | Focal adhesion | 35 | 0.1741 | 5.28E-26 | 7.80E-24 |
| PID_INTEGRIN1_PATHWAY | 66 | Beta1 integrin cell surface interactions | 21 | 0.3182 | 4.81E-22 | 6.39E-20 |
| REACTOME_EXTRACELLULAR_MATRIX_ORGANIZATION | 87 | Genes involved in Extracellular matrix organization | 23 | 0.2644 | 6.13E-22 | 7.41E-20 |
| REACTOME_COLLAGEN_FORMATION | 58 | Genes involved in Collagen formation | 20 | 0.3448 | 7.22E-22 | 8.00E-20 |
| REACTOME_RESPONSE_TO_ELEVATED_PLATELET_CYTOSOLIC_ | 89 | Genes involved in Response to elevated platelet cytosolic Ca2+ | 23 | 0.2584 | 1.09E-21 | 1.10E-19 |
| REACTOME_FATTY_ACID_TRIACYLGLYCEROL_AND_KETONE_BODY_METABOLISM | 168 | Genes involved in Fatty acid, triacylglycerol, and ketone body metabolism | 29 | 0.1726 | 1.16E-21 | 1.10E-19 |
| REACTOME_DEVELOPMENTAL_ | 396 | Genes involved in Developmental Biology | 41 | 0.1035 | 2.45E-21 | 2.17E-19 |
| KEGG_ECM_RECEPTOR_INTERA | 84 | ECM-receptor interaction | 22 | 0.2619 | 6.03E-21 | 5.01E-19 |
| REACTOME_HEMOSTASIS | 466 | Genes involved in Hemostasis | 43 | 0.0923 | 2.21E-20 | 1.73E-18 |
| REACTOME_PLATELET_ACTIVATION_SIGNALING_AND_AGGREG | 208 | Genes involved in Platelet activation, signaling and aggregation | 29 | 0.1394 | 5.29E-19 | 3.91E-17 |
| NABA_COLLAGENS | 44 | Genes encoding collagen proteins | 16 | 0.3636 | 3.38E-18 | 2.37E-16 |
| REACTOME_MUSCLE_CONTRAC | 48 | Genes involved in Muscle contraction | 16 | 0.3333 | 1.73E-17 | 1.15E-15 |
| PID_AVB3_INTEGRIN_PATHWA | 75 | Integrins in angiogenesis | 18 | 0.24 | 1.20E-16 | 7.60E-15 |
| PID_HNF3B_PATHWAY | 45 | FOXA2 and FOXA3 transcription factor networks | 15 | 0.3333 | 1.76E-16 | 1.07E-14 |
| REACTOME_SIGNALING_BY_PD | 122 | Genes involved in Signaling by PDGF | 20 | 0.1639 | 6.45E-15 | 3.73E-13 |
| REACTOME_COMPLEMENT_CA | 32 | Genes involved in Complement cascade | 12 | 0.375 | 3.62E-14 | 2.00E-12 |
| REACTOME_STRIATED_MUSCLE_CONTRACTION | 27 | Genes involved in Striated Muscle Contraction | 11 | 0.4074 | 1.43E-13 | 7.59E-12 |
| REACTOME_TRANSMEMBRANE_TRANSPORT_OF_SMALL_MOL | 413 | Genes involved in Transmembrane transport of small molecules | 32 | 0.0775 | 2.17E-13 | 1.11E-11 |
| REACTOME_METABOLISM_OF_CARBOHYDRATES | 247 | Genes involved in Metabolism of carbohydrates | 25 | 0.1012 | 2.61E-13 | 1.28E-11 |
| PID_INTEGRIN3_PATHWAY | 43 | Beta3 integrin cell surface interactions | 12 | 0.2791 | 2.10E-12 | 9.95E-11 |

| | | | | | | |
|---|---|---|---|---|---|---|
| REACTOME_AXON_GUIDANCE | 251 | Genes involved in Axon guidance | 24 | 0.0956 | 2.67E-12 | 1.22E-10 |
| BIOCARTA_COMP_PATHWAY | 19 | Complement Pathway | 9 | 0.4737 | 4.57E-12 | 2.02E-10 |
| PID_SYNDECAN_1_PATHWAY | 46 | Syndecan-1-mediated signaling events | 12 | 0.2609 | 5.09E-12 | 2.18E-10 |
| KEGG_REGULATION_OF_ACTIN | 216 | Regulation of actin cytoskeleton | 22 | 0.1019 | 6.19E-12 | 2.57E-10 |
| PID_P53_DOWNSTREAM_PATH | 137 | Direct p53 effectors | 18 | 0.1314 | 7.12E-12 | 2.87E-10 |
| REACTOME_PPARA_ACTIVATES_GENE_EXPRESSION | 104 | Genes involved in PPARA Activates Gene Expression | 16 | 0.1538 | 8.98E-12 | 3.51E-10 |
| BIOCARTA_CLASSIC_PATHWAY | 14 | Classical Complement Pathway | 8 | 0.5714 | 1.01E-11 | 3.72E-10 |
| REACTOME_COMMON_PATHW | 14 | Genes involved in Common Pathway | 8 | 0.5714 | 1.01E-11 | 3.72E-10 |
| REACTOME_NCAM1_INTERACTI | 39 | Genes involved in NCAM1 interactions | 11 | 0.2821 | 1.55E-11 | 5.52E-10 |
| REACTOME_IMMUNE_SYSTEM | 933 | Genes involved in Immune System | 46 | 0.0493 | 1.58E-11 | 5.52E-10 |
| BIOCARTA_INTRINSIC_PATHWA | 23 | Intrinsic Prothrombin Activation Pathway | 9 | 0.3913 | 3.82E-11 | 1.30E-09 |
| REACTOME_FORMATION_OF_FIBRIN_CLOT_CLOTTING_CASCA | 32 | Genes involved in Formation of Fibrin Clot (Clotting Cascade) | 10 | 0.3125 | 4.18E-11 | 1.39E-09 |
| REACTOME_METABOLISM_OF_AMINO_ACIDS_AND_DERIVATI | 200 | Genes involved in Metabolism of amino acids and derivatives | 20 | 0.1 | 7.85E-11 | 2.54E-09 |
| REACTOME_LIPID_DIGESTION_MOBILIZATION_AND_TRANSPO | 46 | Genes involved in Lipid digestion, mobilization, and transport | 11 | 0.2391 | 1.11E-10 | 3.52E-09 |
| NABA_PROTEOGLYCANS | 35 | Genes encoding proteoglycans | 10 | 0.2857 | 1.14E-10 | 3.52E-09 |
| REACTOME_LIPOPROTEIN_MET | 28 | Genes involved in Lipoprotein metabolism | 9 | 0.3214 | 3.01E-10 | 9.09E-09 |
| BIOCARTA_AMI_PATHWAY | 20 | Acute Myocardial Infarction | 8 | 0.4 | 3.89E-10 | 1.14E-08 |
| KEGG_CARDIAC_MUSCLE_CON | 80 | Cardiac muscle contraction | 13 | 0.1625 | 3.96E-10 | 1.14E-08 |
| REACTOME_REGULATION_OF_COMPLEMENT_CASCADE | 14 | Genes involved in Regulation of Complement cascade | 7 | 0.5 | 7.30E-10 | 2.06E-08 |
| KEGG_FATTY_ACID_METABOLIS | 42 | Fatty acid metabolism | 10 | 0.2381 | 8.27E-10 | 2.24E-08 |
| PID_UPA_UPAR_PATHWAY | 42 | Urokinase-type plasminogen activator (uPA) and uPAR-mediated signaling | 10 | 0.2381 | 8.27E-10 | 2.24E-08 |
| KEGG_HYPERTROPHIC_CARDIO | 85 | Hypertrophic cardiomyopathy (HCM) | 13 | 0.1529 | 8.64E-10 | 2.30E-08 |
| REACTOME_CELL_SURFACE_INTERACTIONS_AT_THE_VASCULA | 91 | Genes involved in Cell surface interactions at the vascular wall | 13 | 0.1429 | 2.06E-09 | 5.36E-08 |
| REACTOME_INITIAL_TRIGGERING_OF_COMPLEMENT | 16 | Genes involved in Initial triggering of complement | 7 | 0.4375 | 2.37E-09 | 6.05E-08 |
| KEGG_PATHWAYS_IN_CANCER | 328 | Pathways in cancer | 23 | 0.0701 | 3.55E-09 | 8.89E-08 |
| REACTOME_INTEGRIN_CELL_SURFACE_INTERACTIONS | 79 | Genes involved in Integrin cell surface interactions | 12 | 0.1519 | 4.17E-09 | 1.03E-07 |
| REACTOME_NCAM_SIGNALING_FOR_NEURITE_OUT_GROWTH | 64 | Genes involved in NCAM signaling for neurite out-growth | 11 | 0.1719 | 4.80E-09 | 1.16E-07 |
| REACTOME_TCA_CYCLE_AND_RESPIRATORY_ELECTRON_TRANS | 141 | Genes involved in The citric acid (TCA) cycle and respiratory electron transport | 15 | 0.1064 | 7.76E-09 | 1.84E-07 |
| NABA_BASEMENT_MEMBRANES | 40 | Genes encoding structural components of basement membranes | 9 | 0.225 | 1.01E-08 | 2.35E-07 |
| BIOCARTA_FIBRINOLYSIS_PATH | 12 | Fibrinolysis Pathway | 6 | 0.5 | 1.28E-08 | 2.93E-07 |
| REACTOME_GLYCOSAMINOGLYCAN_METABOLISM | 111 | Genes involved in Glycosaminoglycan metabolism | 13 | 0.1171 | 2.42E-08 | 5.44E-07 |
| KEGG_DILATED_CARDIOMYOPA | 92 | Dilated cardiomyopathy | 12 | 0.1304 | 2.46E-08 | 5.44E-07 |
| KEGG_INSULIN_SIGNALING_PA | 137 | Insulin signaling pathway | 14 | 0.1022 | 4.08E-08 | 8.81E-07 |
| KEGG_PEROXISOME | 78 | Peroxisome | 11 | 0.141 | 4.11E-08 | 8.81E-07 |
| KEGG_GLYCOLYSIS_GLUCONEO [illegible] | 62 | Glycolysis / Gluconeogenesis | 10 | 0.1613 | 4.55E-08 | 9.59E-07 |
| REACTOME_GLUCONEOGENESI | 34 | Genes involved in Gluconeogenesis | 8 | 0.2353 | 4.62E-08 | 9.59E-07 |
| PID_HIF1_TFPATHWAY | 66 | HIF-1-alpha transcription factor network | 10 | 0.1515 | 8.44E-08 | 1.73E-06 |
| REACTOME_CHYLOMICRON_MEDIATED_LIPID_TRANSPORT | 16 | Genes involved in Chylomicron-mediated lipid transport | 6 | 0.375 | 1.05E-07 | 2.08E-06 |

| | | | | | | |
|---|---|---|---|---|---|---|
| REACTOME_REGULATION_OF_INSULIN_LIKE_GROWTH_FACTOR_IGF_ACTIVITY_BY_INSULIN_LI | 16 | Genes involved in Regulation of Insulin-like Growth Factor (IGF) Activity by Insulin-like Growth Factor Binding Proteins (IGFBPs) | 6 | 0.375 | 1.05E-07 | 2.08E-06 |
| REACTOME_TRIGLYCERIDE_BIO | 38 | Genes involved in Triglyceride Biosynthesis | 8 | 0.2105 | 1.18E-07 | 2.30E-06 |
| REACTOME_INNATE_IMMUNE_ | 279 | Genes involved in Innate Immune System | 19 | 0.0681 | 1.27E-07 | 2.45E-06 |
| REACTOME_GLUCOSE_METAB | 69 | Genes involved in Glucose metabolism | 10 | 0.1449 | 1.30E-07 | 2.48E-06 |
| REACTOME_TRANSPORT_OF_GLUCOSE_AND_OTHER_SUGARS_BILE_SALTS_AND_ORGANIC_A | 89 | Genes involved in Transport of glucose and other sugars, bile salts and organic acids, metal ions and amine compounds | 11 | 0.1236 | 1.65E-07 | 3.09E-06 |
| KEGG_VIRAL_MYOCARDITIS | 73 | Viral myocarditis | 10 | 0.137 | 2.25E-07 | 4.16E-06 |
| BIOCARTA_PPARA_PATHWAY | 58 | Mechanism of Gene Regulation by Peroxisome Proliferators via PPARa(alpha) | 9 | 0.1552 | 3.04E-07 | 5.54E-06 |
| REACTOME_SLC_MEDIATED_TRANSMEMBRANE_TRANSPORT | 241 | Genes involved in SLC-mediated transmembrane transport | 17 | 0.0705 | 3.53E-07 | 6.27E-06 |
| PID_ATF2_PATHWAY | 59 | ATF-2 transcription factor network | 9 | 0.1525 | 3.54E-07 | 6.27E-06 |
| KEGG_SYSTEMIC_LUPUS_ERYT | 140 | Systemic lupus erythematosus | 13 | 0.0929 | 3.81E-07 | 6.65E-06 |
| REACTOME_KERATAN_SULFATE_DEGRADATION | 11 | Genes involved in Keratan sulfate degradation | 5 | 0.4545 | 4.09E-07 | 7.06E-06 |
| KEGG_ADIPOCYTOKINE_SIGNAL | 67 | Adipocytokine signaling pathway | 9 | 0.1343 | 1.08E-06 | 1.84E-05 |
| BIOCARTA_CBL_PATHWAY | 13 | CBL mediated ligand-induced downregulation of EGF receptors | 5 | 0.3846 | 1.11E-06 | 1.84E-05 |
| BIOCARTA_EXTRINSIC_PATHWA | 13 | Extrinsic Prothrombin Activation Pathway | 5 | 0.3846 | 1.11E-06 | 1.84E-05 |
| REACTOME_DIABETES_PATHW | 133 | Genes involved in Diabetes pathways | 12 | 0.0902 | 1.44E-06 | 2.37E-05 |
| KEGG_CELL_ADHESION_MOLEC | 134 | Cell adhesion molecules (CAMs) | 12 | 0.0896 | 1.56E-06 | 2.50E-05 |
| KEGG_TIGHT_JUNCTION | 134 | Tight junction | 12 | 0.0896 | 1.56E-06 | 2.50E-05 |
| BIOCARTA_PLATELETAPP_PATH | 14 | Platelet Amyloid Precursor Protein Pathway | 5 | 0.3571 | 1.70E-06 | 2.69E-05 |
| REACTOME_TRANSCRIPTIONAL_REGULATION_OF_WHITE_ADI | 72 | Transcriptional Regulation of White Adipocyte Differentiation | 9 | 0.125 | 2.00E-06 | 3.10E-05 |
| KEGG_ARGININE_AND_PROLIN | 54 | Arginine and proline metabolism | 8 | 0.1481 | 2.01E-06 | 3.10E-05 |
| REACTOME_SMOOTH_MUSCLE_CONTRACTION | 25 | Genes involved in Smooth Muscle Contraction | 6 | 0.24 | 2.05E-06 | 3.14E-05 |
| REACTOME_HDL_MEDIATED_LIPID_TRANSPORT | 15 | Genes involved in HDL-mediated lipid transport | 5 | 0.3333 | 2.52E-06 | 3.81E-05 |
| REACTOME_KERATAN_SULFATE_BIOSYNTHESIS | 26 | Genes involved in Keratan sulfate biosynthesis | 6 | 0.2308 | 2.64E-06 | 3.94E-05 |
| KEGG_FC_GAMMA_R_MEDIATE [illegible] | 97 | Fc gamma R-mediated phagocytosis | 10 | 0.1031 | 3.25E-06 | 4.80E-05 |
| PID_AMB2_NEUTROPHILS_PAT | 41 | amb2 Integrin signaling | 7 | 0.1707 | 3.30E-06 | 4.82E-05 |
| PID_INTEGRIN2_PATHWAY | 29 | Beta2 integrin cell surface interactions | 6 | 0.2069 | 5.22E-06 | 7.55E-05 |
| REACTOME_KERATAN_SULFATE_KERATIN_METABOLISM | 30 | Genes involved in Keratan sulfate/keratin metabolism | 6 | 0.2 | 6.44E-06 | 9.12E-05 |
| PID_ENDOTHELIN_PATHWAY | 63 | Endothelins | 8 | 0.127 | 6.59E-06 | 9.12E-05 |
| PID_MYC_REPRESS_PATHWAY | 63 | Validated targets of C-MYC transcriptional | 8 | 0.127 | 6.59E-06 | 9.12E-05 |
| REACTOME_INTERFERON_GAM | 63 | Genes involved in Interferon gamma signaling | 8 | 0.127 | 6.59E-06 | 9.12E-05 |
| PID_CMYB_PATHWAY | 84 | C-MYB transcription factor network | 9 | 0.1071 | 7.31E-06 | 1.00E-04 |
| KEGG_TYPE_II_DIABETES_MELL | 47 | Type II diabetes mellitus | 7 | 0.1489 | 8.51E-06 | 1.15E-04 |
| REACTOME_ACTIVATED_AMPK_STIMULATES_FATTY_ACID_OXI | 19 | Genes involved in Activated AMPK stimulates fatty-acid oxidation in muscle | 5 | 0.2632 | 9.27E-06 | 1.23E-04 |
| REACTOME_PYRUVATE_METAB | 19 | Genes involved in Pyruvate metabolism | 5 | 0.2632 | 9.27E-06 | 1.23E-04 |

| | |
|---|---|
| GSEA parameters: | |
| Collection(s): | Canonical Pathways |
| # overlaps shown: | 100 |
| # genesets in collections: | 1329 |
| # genes in comparison (n): | 724 |
| # genes in universe (N): | 45956 |
