# Supplementary material for: Dietary epicatechin improves survival and delays skeletal muscle degeneration in aged mice
Source: FASEB J. 2018 Aug 10;33(1):965–77. doi: 10.1096/fj.201800554RR (PMC6355074; doi:10.1096/fj.201800554RR)
Supplement: Supplementary file 3 [file fj.201800554RR.st3.pdf]

Supplemental table 3: Top 100 pathways that were associated with epicatechin effects based on 222 genes with significantly differential expression between OC and EC (i.e., OC vs. EC)

Gene Set (Pathway) Name	# Genes in Gene Set (K)	Description	# Genes in Overlap (k)	k/K	p-value	FDR q-value
NABA_CORE_MATRISOME	275	Ensemble of genes encoding core extracellular matrix including ECM glycoproteins, collagens and proteoglycans	20	0.073	1.58E-18	2.10E-15
NABA_MATRISOME	1028	Ensemble of genes encoding extracellular matrix and extracellular matrix-associated proteins	29	0.028	3.03E-15	2.01E-12
REACTOME_DEVELOPMENTAL_BIOLOGY	396	Genes involved in Developmental Biology	19	0.048	2.38E-14	1.05E-11
REACTOME_METABOLISM_OF_LIPIDS_AND_LIPOPROTEINS	478	Genes involved in Metabolism of lipids and lipoproteins	20	0.042	6.23E-14	2.07E-11
NABA_COLLAGENS	44	Genes encoding collagen proteins	9	0.205	3.47E-13	8.96E-11
PID_INTEGRIN1_PATHWAY	66	Beta1 integrin cell surface interactions	10	0.152	4.04E-13	8.96E-11
KEGG_PPAR_SIGNALING_PATHWA	69	PPAR signaling pathway	10	0.145	6.44E-13	1.22E-10
REACTOME_NEURONAL_SYSTEM	279	Genes involved in Neuronal System	15	0.054	2.75E-12	4.44E-10
REACTOME_TRANSMISSION_ACROSS_CHEMICAL_SYNAPSES	186	Genes involved in Transmission across Chemical Synapses	13	0.07	3.01E-12	4.44E-10
KEGG_ECM_RECEPTOR_INTERACTION	84	ECM-receptor interaction	10	0.119	4.94E-12	5.97E-10
REACTOME_COLLAGEN_FORMATION	58	Genes involved in Collagen formation	9	0.155	4.94E-12	5.97E-10
REACTOME_TRANSMEMBRANETRANSPORT_SMALLMOLECULE	413	Genes involved in Transmembrane transport of small molecules	17	0.041	6.59E-12	7.30E-10
REACTOME_SIGNALING_PDGF	122	Genes involved in Signaling by PDGF	11	0.09	9.18E-12	9.38E-10
REACTOME_FATTY_ACID_TRIACYLGLYCEROL_KETONE_BODY	168	Genes involved in Fatty acid, triacylglycerol, and ketone body metabolism	12	0.071	1.63E-11	1.55E-09
REACTOME_GLUCOSE_METABOLI	69	Genes involved in Glucose metabolism	9	0.13	2.52E-11	2.24E-09
KEGG_FOCAL_ADHESION	201	Focal adhesion	12	0.06	1.32E-10	1.10E-08
REACTOME_INTEGRATION_OF_ENERGY_METABOLISM	120	Genes involved in Integration of energy metabolism	10	0.083	1.81E-10	1.41E-08
REACTOME_EXTRACELLULAR_MATRIX_ORGANIZATION	87	Genes involved in Extracellular matrix organization	9	0.103	2.13E-10	1.57E-08
REACTOME_TRIGLYCERIDE_BIOSY	38	Genes involved in Triglyceride Biosynthesis	7	0.184	3.44E-10	2.41E-08
PID_ENDOTHELIN_PATHWAY	63	Endothelins	8	0.127	4.13E-10	2.64E-08
REACTOME_NCAM1_INTERACTIO	39	Genes involved in NCAM1 interactions	7	0.18	4.18E-10	2.64E-08
REACTOME_NCAM_SIGNALINGFOR_NEURITE_OUT_GROWTH	64	Genes involved in NCAM signaling for neurite out-growth	8	0.125	4.70E-10	2.84E-08
KEGG_INSULIN_SIGNALING_PATH	137	Insulin signaling pathway	10	0.073	6.69E-10	3.87E-08
REACTOME_METABOLISM_OF_CARBOHYDRATES	247	Genes involved in Metabolism of carbohydrates	12	0.049	1.40E-09	7.53E-08
PID_SYNDECAN_1_PATHWAY	46	Syndecan-1-mediated signaling events	7	0.152	1.42E-09	7.53E-08
PID_AVB3_INTEGRIN_PATHW	75	Integrins in angiogenesis	8	0.107	1.72E-09	8.78E-08
REACTOME_GLUCONEOGENES	34	Genes involved in Gluconeogenesis	6	0.177	8.62E-09	4.24E-07
REACTOME_NEUROTRANSMITTER_RECEPTOR_BINDING_	137	Genes involved in Neurotransmitter Receptor Binding And Downstream Transmission In The	9	0.066	1.23E-08	5.84E-07
REACTOME_FATTY_ACYL_COA_BIOSYNTHESIS	18	Genes involved in Fatty Acyl-CoA Biosynthesis	5	0.278	1.34E-08	6.15E-07
REACTOME_GABA_B_RECEPTOR_ACTIVATION	38	Genes involved in GABA B receptor activation	6	0.158	1.74E-08	7.72E-07
REACTOME_AXON_GUIDANCE	251	Genes involved in Axon guidance	11	0.044	1.99E-08	8.53E-07
NABA_BASEMENT_MEMBRANES	40	Genes encoding structural components of	6	0.15	2.41E-08	9.99E-07

REACTOME_TRANSCRIPTIONAL_REGULATION_OF_WHITE_ADIPOCY	72	Genes involved in Transcriptional Regulation of White Adipocyte Differentiation	7	0.097	3.53E-08	1.42E-06
PID_INTEGRIN3_PATHWAY	43	Beta3 integrin cell surface interactions	6	0.14	3.78E-08	1.48E-06
REACTOME_GABA_RECEPTOR_AC	52	Genes involved in GABA receptor activation	6	0.115	1.22E-07	4.63E-06
REACTOME_SLC_MEDIATED_TRANSMEMBRANE_TRANSPORT	241	Genes involved in SLC-mediated transmembrane transport	10	0.042	1.48E-07	5.45E-06
REACTOME_HEMOSTASIS	466	Genes involved in Hemostasis	13	0.028	2.03E-07	7.28E-06
REACTOME_TRANSPORT_OF_INORGANIC_CATIONS_ANIONS_AND_	94	Genes involved in Transport of inorganic cations/anions and amino acids/oligopeptides	7	0.075	2.26E-07	7.91E-06
REACTOME_ADENYLATE_CYCLASE_INHIBITORY_PATHWAY	13	Genes involved in Adenylate cyclase inhibitory pathway	4	0.308	2.61E-07	8.68E-06
REACTOME_HORMONE_SENSITIVE_LIPASE_HSL_MEDIATED_TRIACY	13	Genes involved in Hormone-sensitive lipase (HSL)-mediated triacylglycerol hydrolysis	4	0.308	2.61E-07	8.68E-06
NABA_ECM_GLYCOPROTEINS	196	Genes encoding structural ECM glycoproteins	9	0.046	2.68E-07	8.68E-06
REACTOME_PKA_MEDIATED_PHO	18	Genes involved in PKA-mediated phosphorylation	4	0.222	1.10E-06	3.48E-05
REACTOME_G_ALPHA_S_SIGNALLI	121	Genes involved in G alpha (s) signalling events	7	0.058	1.26E-06	3.90E-05
REACTOME_PLC_BETA_MEDIATED	43	Genes involved in PLC beta mediated events	5	0.116	1.38E-06	4.16E-05
REACTOME_INTEGRIN_CELL_SURFACE_INTERACTIONS	79	Genes involved in Integrin cell surface interactions	6	0.076	1.51E-06	4.46E-05
REACTOME_G_ALPHA_Z_SIGNALLING_EVENTS	44	Genes involved in G alpha (z) signalling events	5	0.114	1.55E-06	4.47E-05
REACTOME_LIPID_DIGESTION_MOBILIZATION_AND_TRANSPORT	46	Genes involved in Lipid digestion, mobilization, and transport	5	0.109	1.94E-06	5.49E-05
REACTOME_AMINO_ACID_AND_OLIGOPEPTIDE_SLC_TRANSPORTER	49	Genes involved in Amino acid and oligopeptide SLC transporters	5	0.102	2.67E-06	7.39E-05
REACTOME_AQUAPORIN_MEDIATED_TRANSPORT	51	Genes involved in Aquaporin-mediated transport	5	0.098	3.27E-06	8.86E-05
BIOCARTA_NOS1_PATHWAY	24	Nitric Oxide Signaling Pathway	4	0.167	3.74E-06	9.93E-05
REACTOME_PHOSPHOLIPASE_C_MEDIATED_CASCADE	54	Genes involved in Phospholipase C-mediated cascade	5	0.093	4.35E-06	1.13E-04
KEGG_PENTOSE_PHOSPHATE_PAT	27	Pentose phosphate pathway	4	0.148	6.11E-06	1.56E-04
REACTOME_GLYCOLYSIS	29	Genes involved in Glycolysis	4	0.138	8.21E-06	2.06E-04
KEGG_GLYCOLYSIS_GLUCONEOGE	62	Glycolysis / Gluconeogenesis	5	0.081	8.65E-06	2.13E-04
REACTOME_CA_DEPENDENT_EVE	30	Genes involved in Ca-dependent events	4	0.133	9.44E-06	2.28E-04
BIOCARTA_GLYCOLYSIS_PATHWA	10	Glycolysis Pathway	3	0.3	1.01E-05	2.36E-04
REACTOME_ADENYLATE_CYCLASE_ACTIVATING_PATHWAY	10	Genes involved in Adenylate cyclase activating pathway	3	0.3	1.01E-05	2.36E-04
PID_LYSOPHOSPHOLIPID_PATHW	66	LPA receptor mediated events	5	0.076	1.18E-05	2.70E-04
REACTOME_DAG_AND_IP3_SIGNA	32	Genes involved in DAG and IP3 signaling	4	0.125	1.23E-05	2.77E-04
REACTOME_GLUCAGON_SIGNALING_IN_METABOLIC_REGULATION	34	Genes involved in Glucagon signaling in metabolic regulation	4	0.118	1.58E-05	3.49E-04
KEGG_CALCIUM_SIGNALING_PAT	178	Calcium signaling pathway	7	0.039	1.61E-05	3.51E-04
BIOCARTA_GPCR_PATHWAY	37	Signaling Pathway from G-Protein Families	4	0.108	2.22E-05	4.68E-04
BIOCARTA_PAR1_PATHWAY	37	Thrombin signaling and protease-activated	4	0.108	2.22E-05	4.68E-04
REACTOME_OPIOID_SIGNALLING	78	Genes involved in Opioid Signalling	5	0.064	2.66E-05	5.53E-04
PID_LPA4_PATHWAY	15	LPA4-mediated signaling events	3	0.2	3.77E-05	7.71E-04
KEGG_PROGESTERONE_MEDIATED_OOCYTE_MATURATION	86	Progesterone-mediated oocyte maturation	5	0.058	4.27E-05	8.59E-04
REACTOME_PLATELET_ACTIVATION_SIGNALING_AND_AGGREGATIO	208	Genes involved in Platelet activation, signaling and aggregation	7	0.034	4.36E-05	8.64E-04
REACTOME_REGULATION_OF_WATER_BALANCE_BY_RENAL_AQUAP	44	Genes involved in Regulation of Water Balance by Renal Aquaporins	4	0.091	4.45E-05	8.70E-04
PID_HNF3B_PATHWAY	45	FOXA2 and FOXA3 transcription factor networks	4	0.089	4.87E-05	9.38E-04
KEGG_GAP_JUNCTION	90	Gap junction	5	0.056	5.31E-05	1.01E-03

REACTOME_REGULATION_OF_INSULIN_SECRETION	93	Genes involved in Regulation of Insulin Secretion	5	0.054	6.21E-05	1.16E-03
KEGG_GLYCEROLIPID_METABOLIS	49	Glycerolipid metabolism	4	0.082	6.83E-05	1.26E-03
REACTOME_DOWNSTREAM_SIGNALING_ACTIVATED_FGFR	100	Genes involved in Downstream signaling of activated FGFR	5	0.05	8.78E-05	1.60E-03
KEGG_GNRH_SIGNALING_PATHW	101	GnRH signaling pathway	5	0.05	9.20E-05	1.65E-03
KEGG_MELANOGENESIS	102	Melanogenesis	5	0.049	9.64E-05	1.71E-03
REACTOME_PPARA_ACTIVATES_GENE_EXPRESSION	104	Genes involved in PPARA Activates Gene Expression	5	0.048	1.06E-04	1.85E-03
KEGG_PATHWAYS_IN_CANCER	328	Pathways in cancer	8	0.024	1.19E-04	2.06E-03
KEGG_BIOSYNTHESIS_OF_UNSATURATED_FATTY_ACIDS	22	Biosynthesis of unsaturated fatty acids	3	0.136	1.25E-04	2.13E-03
KEGG_PROXIMAL_TUBULE_BICARBONATE_RECLAMATION	23	Proximal tubule bicarbonate reclamation	3	0.13	1.43E-04	2.40E-03
REACTOME_SIGNALING_FGFR	112	Genes involved in Signaling by FGFR	5	0.045	1.50E-04	2.49E-03
BIOCARTA_CXCR4_PATHWAY	24	CXCR4 Signaling Pathway	3	0.125	1.63E-04	2.64E-03
REACTOME_SULFUR_AMINO_ACID_METABOLISM	24	Genes involved in Sulfur amino acid metabolism	3	0.125	1.63E-04	2.64E-03
KEGG_VASCULAR_SMOOTH_MUSCLE_CONTRACTION	115	Vascular smooth muscle contraction	5	0.044	1.70E-04	2.71E-03
REACTOME_INHIBITION_OF_INSULIN_SECRETION_BY_ADRENALINE_	25	Genes involved in Inhibition of Insulin Secretion by Adrenaline/Noradrenaline	3	0.12	1.84E-04	2.92E-03
KEGG_CHEMOKINE_SIGNALING_PATHWAY	190	Chemokine signaling pathway	6	0.032	2.18E-04	3.39E-03
PID_HIF1_TFPATHWAY	66	HIF-1-alpha transcription factor network	4	0.061	2.19E-04	3.39E-03
KEGG_NEUROACTIVE_LIGAND_RECEPTOR_INTERACTION	272	Neuroactive ligand-receptor interaction	7	0.026	2.30E-04	3.48E-03
BIOCARTA_CREB_PATHWAY	27	Transcription factor CREB and its extracellular	3	0.111	2.33E-04	3.48E-03
BIOCARTA_EDG1_PATHWAY	27	Phospholipids as signalling intermediaries	3	0.111	2.33E-04	3.48E-03
REACTOME_SIGNALING_GPCR	920	Genes involved in Signaling by GPCR	13	0.014	2.65E-04	3.91E-03
REACTOME_SIGNALING_BY_FGFR_IN_DISEASE	127	Genes involved in Signaling by FGFR in disease	5	0.039	2.69E-04	3.93E-03
KEGG_LONG_TERM_POTENTIATIO	70	Long-term potentiation	4	0.057	2.75E-04	3.97E-03
REACTOME_GPCR_DOWNSTREAM_SIGNALING	805	Genes involved in GPCR downstream signaling	12	0.015	2.82E-04	4.03E-03
KEGG_GLYCINE_SERINE_AND_THREONINE_METABOLISM	31	Glycine, serine and threonine metabolism	3	0.097	3.53E-04	5.00E-03
REACTOME_NGF_SIGNALLING_VIA_TRKA_FROM_THE_PLASMA_ME	137	Genes involved in NGF signalling via TRKA from the plasma membrane	5	0.037	3.81E-04	5.34E-03
KEGG_PROPANOATE_METABOLIS	33	Propanoate metabolism	3	0.091	4.26E-04	5.84E-03
REACTOME_POST_NMDA_RECEPTOR_ACTIVATION_EVENTS	33	Genes involved in Post NMDA receptor activation events	3	0.091	4.26E-04	5.84E-03
REACTOME_SIGNALLING_NGF	217	Genes involved in Signalling by NGF	6	0.028	4.43E-04	6.00E-03
BIOCARTA_MPR_PATHWAY	34	How Progesterone Initiates Oocyte Membrane	3	0.088	4.66E-04	6.13E-03
KEGG_BUTANOATE_METABOLISM	34	Butanoate metabolism	3	0.088	4.66E-04	6.13E-03

Collection(s):	Canonical Pathways
# overlaps shown:	100
# genesets in collections:	1329
# genes in comparison (n):	204
# genes in universe (N):	45956
