# Supplementary material for: Dietary epicatechin improves survival and delays skeletal muscle degeneration in aged mice
Source: FASEB J. 2018 Aug 10;33(1):965–77. doi: 10.1096/fj.201800554RR (PMC6355074; doi:10.1096/fj.201800554RR)
Supplement: Supplementary file 4 [file fj.201800554RR.st4.pdf]

Supplemental table 4: Top 55 pathways that were associated with epicatechin effects based on 92 genes with significantly differential expression in both YC vs. OC and OC vs. EC.

Gene Set (Pathway) Name	# Genes in Gene Set (K)	Description	# Genes in Overlap (k)	k/K	p-value	FDR q-value
NABA_CORE_MATRISOME	275	Ensemble of genes encoding core extracellular matrix including ECM glycoproteins, collagens and proteoglycans	18	0.0655	1.14E-22	1.52E-19
NABA_MATRISOME	1028	Ensemble of genes encoding extracellular matrix and extracellular matrix-associated	23	0.0224	2.51E-18	1.67E-15
PID_INTEGRIN1_PATHW	66	Beta1 integrin cell surface interactions	10	0.1515	8.50E-17	3.76E-14
KEGG_ECM_RECEPTOR_INTERACTION	84	ECM-receptor interaction	10	0.119	1.08E-15	3.59E-13
REACTOME_DEVELOPMENTAL_BIOLOGY	396	Genes involved in Developmental Biology	15	0.0379	1.76E-15	4.68E-13
NABA_COLLAGENS	44	Genes encoding collagen proteins	8	0.1818	2.40E-14	5.31E-12
KEGG_FOCAL_ADHESION	201	Focal adhesion	11	0.0547	2.31E-13	4.22E-11
REACTOME_COLLAGEN_	58	Genes involved in Collagen formation	8	0.1379	2.54E-13	4.22E-11
KEGG_PPAR_SIGNALING	69	PPAR signaling pathway	8	0.1159	1.09E-12	1.56E-10
REACTOME_NCAM1_INT	39	Genes involved in NCAM1 interactions	7	0.1795	1.17E-12	1.56E-10
REACTOME_EXTRACELLULAR_MATRIX_ORGANIZA	87	Genes involved in Extracellular matrix organization	8	0.092	7.39E-12	8.93E-10
REACTOME_NCAM_SIGNALING_FOR_NEURITE_O	64	Genes involved in NCAM signaling for neurite out-growth	7	0.1094	4.56E-11	5.05E-09
REACTOME_SIGNALING_	122	Genes involved in Signaling by PDGF	8	0.0656	1.15E-10	1.18E-08
PID_INTEGRIN3_PATHW	43	Beta3 integrin cell surface interactions	6	0.1395	2.56E-10	2.43E-08
PID_SYNDECAN_1_PATH	46	Syndecan-1-mediated signaling events	6	0.1304	3.91E-10	3.47E-08
REACTOME_AXON_GUID	251	Genes involved in Axon guidance	9	0.0359	1.63E-09	1.35E-07
NABA_ECM_GLYCOPROT	196	Genes encoding structural ECM glycoproteins	8	0.0408	4.98E-09	3.90E-07
PID_AVB3_INTEGRIN_PA	75	Integrins in angiogenesis	6	0.08	8.04E-09	5.94E-07
REACTOME_INTEGRIN_CELL_SURFACE_INTERACTI	79	Genes involved in Integrin cell surface interactions	6	0.0759	1.10E-08	7.71E-07
NABA_BASEMENT_MEM	40	Genes encoding structural components of	5	0.125	1.52E-08	1.01E-06
REACTOME_METABOLISM_OF_LIPIDS_AND_LIPO	478	Genes involved in Metabolism of lipids and lipoproteins	10	0.0209	3.30E-08	2.09E-06
REACTOME_TRANSCRIPTIONAL_REGULATION_OF_WHITE_ADIPOCYTE_DIF	72	Genes involved in Transcriptional Regulation of White Adipocyte Differentiation	5	0.0694	3.07E-07	1.85E-05
REACTOME_FATTY_ACID_TRIACYLGLYCEROL_AND_KETONE_BODY_METAB	168	Genes involved in Fatty acid, triacylglycerol, and ketone body metabolism	6	0.0357	9.87E-07	5.70E-05
REACTOME_TRANSMEMBRANE_TRANSPORT_OF_	413	Genes involved in Transmembrane transport of small molecules	8	0.0194	1.49E-06	8.24E-05
PID_HNF3B_PATHWAY	45	FOXA2 and FOXA3 transcription factor	4	0.0889	1.84E-06	9.79E-05
REACTOME_PPARA_ACTIVATES_GENE_EXPRESSIO	104	Genes involved in PPARA Activates Gene Expression	5	0.0481	1.92E-06	9.83E-05
KEGG_INSULIN_SIGNALING_PATHWAY	137	Insulin signaling pathway	5	0.0365	7.43E-06	3.66E-04

REACTOME_GLUCOSE_M	69	Genes involved in Glucose metabolism	4	0.058	1.03E-05	4.90E-04
KEGG_SMALL_CELL_LUN	84	Small cell lung cancer	4	0.0476	2.25E-05	1.03E-03
REACTOME_HEMOSTASI	466	Genes involved in Hemostasis	7	0.015	3.55E-05	1.57E-03
REACTOME_GLUCONEO	34	Genes involved in Gluconeogenesis	3	0.0882	4.02E-05	1.72E-03
KEGG_PATHWAYS_IN_CA	328	Pathways in cancer	6	0.0183	4.46E-05	1.85E-03
REACTOME_LIPID_DIGESTION_MOBILIZATION_AN	46	Genes involved in Lipid digestion, mobilization, and transport	3	0.0652	1.00E-04	4.04E-03
REACTOME_METABOLISM_OF_CARBOHYDRATES	247	Genes involved in Metabolism of carbohydrates	5	0.0202	1.24E-04	4.84E-03
BIOCARTA_VITCB_PATH	11	Vitamin C in the Brain	2	0.1818	2.02E-04	7.66E-03
REACTOME_PLATELET_ADHESION_TO_EXPOSED_	12	Genes involved in Platelet Adhesion to exposed collagen	2	0.1667	2.42E-04	8.73E-03
KEGG_GLYCOLYSIS_GLUCONEOGENESIS	62	Glycolysis / Gluconeogenesis	3	0.0484	2.44E-04	8.73E-03
PID_ENDOTHELIN_PATH	63	Endothelins	3	0.0476	2.56E-04	8.73E-03
PID_MYC_REPRESS_PAT	63	Validated targets of C-MYC transcriptional	3	0.0476	2.56E-04	8.73E-03
BIOCARTA_ACE2_PATHW	13	Angiotensin-converting enzyme 2 regulates	2	0.1538	2.85E-04	9.25E-03
REACTOME_HORMONE_SENSITIVE_LIPASE_HSL_MEDIATED_TRIACYLGLYC	13	Genes involved in Hormone-sensitive lipase (HSL)-mediated triacylglycerol hydrolysis	2	0.1538	2.85E-04	9.25E-03
BIOCARTA_PLATELETAPP	14	Platelet Amyloid Precursor Protein Pathway	2	0.1429	3.32E-04	1.05E-02
BIOCARTA_COMP_PATH	19	Complement Pathway	2	0.1053	6.21E-04	1.92E-02
BIOCARTA_AMI_PATHW	20	Acute Myocardial Infarction	2	0.1	6.89E-04	2.03E-02
ST_WNT_CA2_CYCLIC_GMP_PATHWAY	20	Wnt/Ca2+/cyclic GMP signaling.	2	0.1	6.89E-04	2.03E-02
REACTOME_PLATELET_ACTIVATION_SIGNALING_	208	Genes involved in Platelet activation, signaling and aggregation	4	0.0192	7.37E-04	2.13E-02
BIOCARTA_INTRINSIC_PA	23	Intrinsic Prothrombin Activation Pathway	2	0.087	9.14E-04	2.53E-02
KEGG_PROXIMAL_TUBULE_BICARBONATE_RECLA	23	Proximal tubule bicarbonate reclamation	2	0.087	9.14E-04	2.53E-02
PID_LYMPH_ANGIOGENESIS_PATHWAY	25	VEGFR3 signaling in lymphatic endothelium	2	0.08	1.08E-03	2.93E-02
KEGG_PENTOSE_PHOSPHATE_PATHWAY	27	Pentose phosphate pathway	2	0.0741	1.26E-03	3.31E-02
REACTOME_SLC_MEDIATED_TRANSMEMBRANE_T	241	Genes involved in SLC-mediated transmembrane transport	4	0.0166	1.27E-03	3.31E-02
REACTOME_GLYCOLYSIS	29	Genes involved in Glycolysis	2	0.069	1.46E-03	3.72E-02
REACTOME_GPVI_MEDIATED_ACTIVATION_CASCA	31	Genes involved in GPVI-mediated activation cascade	2	0.0645	1.66E-03	4.17E-02
REACTOME_G_ALPHA_S_SIGNALLING_EVENTS	121	Genes involved in G alpha (s) signalling events	3	0.0248	1.71E-03	4.22E-02
KEGG_FRUCTOSE_AND_MANNOSE_METABOLIS	34	Fructose and mannose metabolism	2	0.0588	2.00E-03	4.83E-02

Collection(s):	Canonical Pathways
# overlaps shown:	55
# genesets in collections:	1329
# genes in comparison (n)	89
# genes in universe (N):	45956
